# The Lesson Learned from the Unique Evolutionary Story of Avirulence Gene *AvrPii* of *Magnaporthe oryzae*

**DOI:** 10.3390/genes14051065

**Published:** 2023-05-11

**Authors:** Xing Wang, Weihuai Wu, Yaling Zhang, Cheng Li, Jinyan Wang, Jianqiang Wen, Shulin Zhang, Yongxiang Yao, Weisheng Lu, Zhenghong Zhao, Jiasui Zhan, Qinghua Pan

**Affiliations:** 1 Rice Blast Research Center, South China Agricultural University, Guangzhou 510642, China; wangxing8714@126.com (X.W.); weihuaiwu2002@163.com (W.W.); byndzyl@163.com (Y.Z.); licheng_scau@126.com (C.L.); oryza@stu.scau.edu.cn (J.W.); wenjianqiang51@126.com (J.W.); zhangsl80h@163.com (S.Z.); yaoyongxiang0415@163.com (Y.Y.); lws2869@scau.edu.cn (W.L.); 2 Hainan Key Laboratory for Monitoring and Control of Tropical Agricultural Pests, Environment and Plant Protection Institute, Chinese Academy of Tropical Agricultural Sciences, Haikou 571101, China; 3 College of Agronomy, Heilongjiang Bayi Agricultural University, Daqing 163319, China; 4 Department of Plant Pathology, College of Plant Protection, Anhui Agricultural University, Hefei 230036, China; 5 Corn Research Institute, Dandong Academy of Agricultural Sciences, Dandong 118109, China; 6 State Key Laboratory of Hybrid Rice, Rice Research Institute, Hunan Academy of Agricultural Sciences, Changsha 410125, China; huwenbin2007@163.com; 7 Department of Forest Mycology and Plant Pathology, Swedish University of Agricultural Sciences, 75007 Uppsala, Sweden; jiasui.zhan@slu.se

**Keywords:** *Magnaporthe oryzae*, avirulence gene, divergence, haplotype, population structure

## Abstract

Blast, caused by *Magnaporthe oryzae*, is one of the most destructive diseases affecting rice production. Understanding population dynamics of the pathogen’s avirulence genes is pre-required for breeding and then deploying new cultivars carrying promising resistance genes. The divergence and population structure of *AvrPii* was dissected in the populations of southern (Guangdong, Hunan, and Guizhou) and northern (Jilin, Liaoning, and Heilongjiang) China, via population genetic and evolutionary approaches. The evolutionary divergence between a known haplotype *AvrPii*-J and a novel one *AvrPii*-C was demonstrated by haplotype-specific amplicon-based sequencing and genetic transformation. The different avirulent performances of a set of seven haplotype-chimeric mutants suggested that the integrity of the full-length gene structures is crucial to express functionality of individual haplotypes. All the four combinations of phenotypes/genotypes were detected in the three southern populations, and only two in the northern three, suggesting that genic diversity in the southern region was higher than those in the northern one. The population structure of the *AvrPii* family was shaped by balancing, purifying, and positive selection pressures in the Chinese populations. The *AvrPii*-J was recognized as the wild type that emerged before rice domestication. Considering higher frequencies of avirulent isolates were detected in Hunan, Guizhou, and Liaoning, the cognate resistance gene *Pii* could be continuously used as a basic and critical resistance resource in such regions. The unique population structures of the *AvrPii* family found in China have significant implications for understanding how the *AvrPii* family has kept an artful balance and purity among its members (haplotypes) those keenly interact with *Pii* under gene-for-gene relationships. The lesson learned from case studies on the *AvrPii* family is that much attention should be paid to haplotype divergence of target gene.

## 1. Introduction 

Rice (*Oryza sativa* L.), domesticated into subspecies *Xian* (*indica*) and *Geng* (*japonica*), is one of the top three cereal crops in China in terms of area and production [1,2]. Rice blast, caused by the fungal pathogen *M*. *oryzae* (*Mo*; syn. *Pyricularia oryzae*), is the most concerned and worried disease in both Southern *Xian* and Northern *Geng* cropping areas [2,3,4,5]. Like other diseases, it is believed that genetic resistance is the most critical and promising approach to control rice blast, but the effectiveness and durability of resistance depends on population dynamics for the pathogen as well as the host in each region [3,6,7,8]. 

The gene-for-gene principle, in which the pathogen carries a gene for avirulence (*Avr* gene) that corresponds to each resistance gene (*R* gene) in the cognate host, plays a pivotal role in shaping population structures of plant pathogens [2,5,8,9,10,11,12]. The *AvrPii* of *Mo*, which was isolated through association genetic approach (AB498874) [13], was one of the most attractive *Avr* genes being widely investigated in various populations. Xing et al. [14] characterized 182 isolates of *Mo*, which were collected from Hunan, China, by rice monogenic lines-based pathogenicity assay and PCR amplification with gene-specific primer sets. The results showed that among 22.5% of *Pii*-avirulent isolates only 2.5% gained *AvrPii*-amplicons. The distribution and mutation of *AvrPii* in Yunnan, China, was also studied [3]. Out of 454 isolates tested, 291 were *Pii*-virulent. Among them, 24 *Pii*-virulent isolates gained *AvrPii*-specific amplicons, suggesting that the *AvrPii* carried by the 24 isolates has lost avirulence function through smaller variations such as base substitution. Conversely, 105 out of the 163 were *Pii*-avirulent isolates had no *AvrPii*-specific amplicon, indicating that two pairs of *AvrPii*-specific primers used in the investigation might not be suitable for these isolates, and/or avirulent performance of such isolates against the *Pii*-carrier conveyed by another gene pair of *Avr*/*R* genes. In the characterization of 77 isolates collected from Thailand, only 46 isolates successfully achieved coding sequence (CDS) amplification by *AvrPii*-specific primer pair. The CDS of the *AvrPii* gene from the 46 isolates displayed three different haplotypes with 11 nucleotide polymorphic sites, including one major haplotype (44 out of 46 isolates) and two minor haplotypes (one a single isolate each) [11]. The four *Avr* genes, *AvrPita1*, *AvrPik*, *AvrPiz-t*, *AvrPia*, and *AvrPii*, were surveyed in a *Mo* population consisting of 258 isolates collected during 1975–2009 from Arkansas state, the United States. PCR products were obtained from 232 isolates, and the remaining 26 isolates without any amplicon might be due to absence of the *Avr* genes tested, and/or to less matching of *Avr* gene-specific primer sets used in the study. It was surprised that only one isolate carried *AvrPii*, compared to 225 for *AvrPiz-t*, and 174 for *AvrPita1* [15]. Altogether, there was a certain numerical gap between a higher rate of *Pii*-avirulent isolates and a lower rate of *AvrPii*-specific amplicons in the *Mo* population tested. 

Our aim in this study was to investigate whether the main reason for the numerical gap was a higher proportion of sequence-distinct haplotypes in the *AvrPii* family that failed to be amplified by primers designed from the reference isolate. If as hypothesized, how was the *AvrPii* family featured with divergent haplotypes in the *Mo* population? 

## 2. Materials and Methods

### 2.1. Discovery of a New Haplotype AvrPii-C 

Ten isolates (CHL22, 346, 584, 590, 624, EHL0314, 0317, 0319, 0329, 0445) that were avirulent on a rice monogenic line carrying *Pii*, IRBLi-F5, were selected from various *Mo* populations in China and were subjected to amplification of both coding sequence (CDS) and the full-length (FL) sequence of *AvrPii* via the regular polymerase chain reaction (PCR) system, with the key primer pairs AvrPii-J/C-CDS_F/R, and AvrPii-J-FL_F/R, respectively. Both primer pairs were designed based on the known reference sequence of *AvrPii* aforementioned. Because half of the ten isolates tested did not produce any amplicon with the FL primer pair, the alternative approach called a thermal asymmetric interlaced PCR (Tail-PCR) system was employed for creating specific sequences of both 5′ and 3′ regions of the representative isolate, CHL346; both might be distinct from the reference sequence (Figure S1). After three rounds of Tail-PCR, the certain amplicons derived from 5′ and 3′ regions were subjected to sequencing. Then, the key primer pair, AvrPii-C-FL_F/R, was devised based on both sequences for re-amplifying the target gene of the ten isolates via the regular PCR system. The full-length sequences of the ten isolates were compared using the Multalin website (http://multalin.toulouse.inra.fr/multalin/). Information on the primers and PCR programs were shown in Table S1.

### 2.2. Functional Validation of Both Haplotypes 

To construct transgenic vectors of both haplotypes, the full-length sequences of *AvrPii*-C (as that was firstly discovered from the Chinese isolates in the present study) and *AvrPii*-J (as it was firstly discovered from the Japanese isolate Ina168) [13], respectively, were re-amplified using Q5 High-Fidelity DNA polymerase (Tsingke, Nanjing, China) based on the genomic DNA of isolates CHL346 and CHL22. The amplicons were inserted into pGEM-T (Promega, Dalian, China) to generate two constructs pGEM-T_*AvrPii*-C and _*AvrPii*-J. Following their amplification from the pGEM-T templates with the key primer pairs, *AvrPii*-C-GT_F/R (GT, genetic transformation) and *AvrPii*-J-GT_F/R, the transgene sequences were introduced into the binary vector pBHT2-AscI with an *Asc* I recognition site (Table S1) [7,8]. Before their transformation into a virulent recipient isolate CHL357, each construct was validated by sequencing. At least five independent hygromycin-resistant transformants per construct were carried forward for testing their functionality onto the *Pii* carrier, IRBLi-F5, and their specificity onto four monogenic lines carrying individual resistance genes, i.e., IRBL5-M (*Pi5*), IRBLk-Ka (*Pik*), IRBLb-B (*Pib*), and IRBLta-CP1 (*Pita*) [16]. The phenotype of each isolate was scored as A (0 reaction: avirulent), MA (1 to 2 reaction: moderately avirulent), MV (3 to 4 reaction: moderately virulent), and V (5 reaction: virulent) through the routine spraying inoculation procedure as described previously [2,5,17]. 

### 2.3. Construction and Characterization of Haplotype-Chimeric Mutant 

Because multiple variations were scattered in the whole sequences of both haplotypes (Figure S2), the haplotype-chimeric mutants were constructed via a domain-swapping approach (Figure S3) [8]. To create the two haplotype-chimeric mutants in 5′ and 3′ regions, two chimeric templates were amplified with respective primer pairs, with the outside one being haplotype-specific (pure color arrow) and the inside one being haplotype-chimeric (chimeric color arrow); then the haplotype-chimeric mutants were generated by overlap-PCR with the pre-amplified fragment, both serving as megaprimers, for overlap extension. To create three haplotype-chimeric mutants targeted in the CDS region, self-ligation-PCR was conducted with a set of laddering chimeric primers using the TaKaRa MutanBEST Kit (TaKaRa, Dalian, China), according to the manufacturer’s protocol (Table S1 and Figure S3). 

Seven haplotype-chimeric mutants (Mai1-7) with two donor and one recipient isolates were phenotyped as aforementioned. Then the repeated and typical phenotype of each mutant was quantified based on *Mo* growth in the leaves of IRBLi-F5 by estimating the ratio between the copy number of a *Mo* sequence (*Po2*) and the rice *Ubi* gene (GenBank accession D12629) with qPCR system as described previously [8]. To establish relationships (if any) between phenotypes and protein structures of the haplotype-chimeric mutants, two models were employed to draw their 3D-structures (I-TASSER, https://zhanggroup.org/I-TASSER/; SWISS-MODEL, https://swissmodel.expasy.org/interactive) (Figure S4). 

Interactions of the wild types and three CDS derived haplotype-chimeric mutants with the paired Pii proteins were tested in the yeast two-hybrid (Y2H) system (Clontech, Dalian, China), in which the mature (20–70) and the full-length (FL) AvrPii proteins with the five versions, i.e., FL, CC (coiled coil), NBS (nucleotide binding site), LRR (leucine rich repeat), CtNL (carbon terminal non-LRR sequence), of the paired Pii protein (coded as #1, #2) were in reciprocal bait/prey combinations (Figure S5). The detailed procedure was adopted from the previous studies [7,18]. 

### 2.4. Shape of Population Structure

Six *Mo* populations ((Guangdong (GD), Hunan (HN), and Guizhou (GZ) from southern; and Liaoning (LN), Jilin (JL), and Heilongjiang (HLJ) from northern China)), each consisting of 60 rice-derived isolates, were selected based on regionally representative rice cultivars and sites sampled (Table S2). An additional set of 17 weed-derived *Mo* isolates was used as outgroup for checking the origins of haplotypes. A total of 377 isolates were phenotyped on the *Pii* carrier as aforementioned. The isolates were genotyped with four markers, each two, a shorter fragment (MK1) tending to CDS, and a longer one (MK2) to 5′ region for the individual haplotypes, by the key PCR primer pairs, AvrPii-C-MK1_F/R, AvrPii-C-MK2_F/R, AvrPii-J-MK1_F/R, and AvrPii-J-MK2_F/R. Each marker was given a genotype code of either 0 (absence of amplicon) or 1 (presence of amplicon). Then, the genotype of each isolate was simply scored as 0 (absence of *AvrPii*), 1-C (*AvrPii*-C), 1-J (*AvrPii*-J), and 1-C_J (*AvrPii*-C and *AvrPii*-J), when genotypes of each two markers for the individual haplotype were consistent. Taken together, the parameter of each combination of phenotype/genotype was counted out for each population for shaping its population structure. 

### 2.5. Resequencing and Evolution Analysis

All the isolates with amplicons including weed-derived *Mo* isolates were re-amplified by the key primer pairs, AvrPii-C-RS_F/R (RS, resequencing), and/or AvrPii-J-RS_F/R. Particularly, an isolate belonging to genotype 1-C/J might gain two amplicons by both key primer pairs. Then all the amplicons carrying the FL sequences were subjected to resequencing, directly. The *AvrPii* allelic sequences were aligned with the known reference sequence of isolate Ina168 using DnaSP v6.12.03 software (http://www.ub.edu/dnasp/), and their CDSs were subjected to evolutionary analysis with the following parameters. Segregating site (*S*) was derived from pair comparison among isolates tested. Nucleotide diversity (*π*) was estimated by Nei’s function with the Jukes and Cantor correction [19,20]. Selection on the family was evaluated by the Fu and Li’s D* and F* parameters [21], and the average rate of non-synonymous (*Ka*) and synonymous (*Ks*) substitutions (*Ka*/*Ks*) was assessed as described by Nei and Gojobori [22]. Then, three kinds of key selection forces were briefly defined: the positive selection (also defined as Darwinian selection or directional selection) is featured with key evolutionary parameters, *D* < 0 and/or *Ka*/*Ks* (*dN*/*dS*) > 1, that leads to fixation of target allele that increases the fitness of individuals; the balancing selection (disruptive selection or diversified selection) is featured with key parameter, *D* > 0, that favors diversity; and the purifying selection (negative or stabilizing selection) is featured with key parameter, *Ka*/*Ks* ≤ 1, that eliminates deleterious mutations [23,24,25].

For deeply dissecting evolutionary features of the *AvrPii* family, six *Mo* natural populations were recombined into four comparable groups, i.e., all *AvrPii* group, haplotype *AvrPii*-J group, haplotype *AvrPii*-C group, and a regional group. The target genes were further divided into the entire coding region, non-signal peptide region (mature protein), and signal peptide region, if necessary. Additionally, 17 weed-derived isolates served as an outgroup for evolutionary comparison, if any. 

## 3. Results

### 3.1. Haplotypic Features of AvrPii

**Figure 1:**
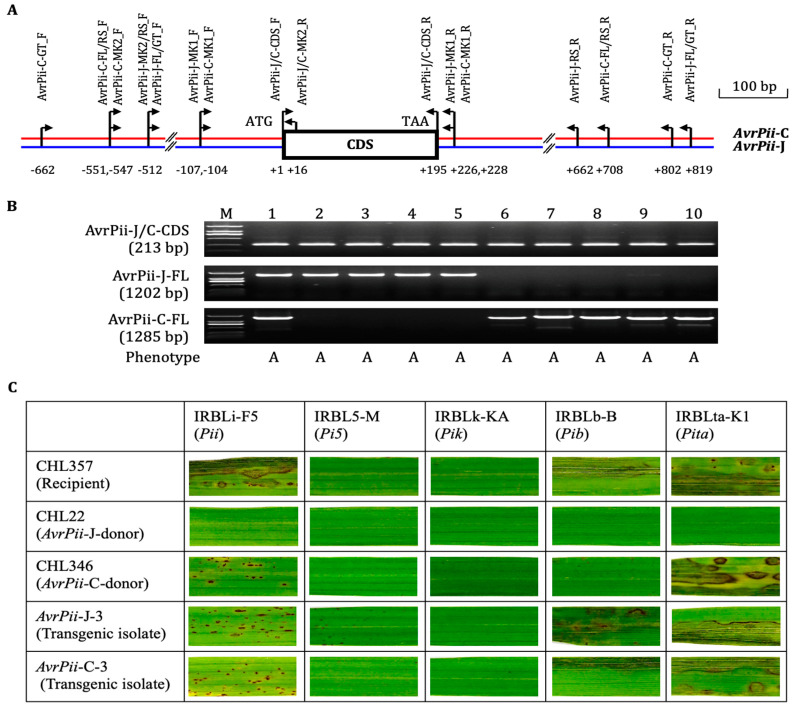
Discovery of a novel haplotype *AvrPii*-C in Chinese populations of *M*. *oryzae*. (**A**) The scheme map of *AvrPii* and the key PCR primer pairs for genotyping, genetic transformation, and re-sequencing of both haplotypes of *AvrPii*. (**B**) Detection of *AvrPii* of 10 *Pii*-avirulent isolates selected from the Chinese populations using a set of three key primer pairs; both AvrPii-J/C-CDS_F/R and AvrPii-J-FL_F/R were adopted from the reference sequence of the Japanese isolate, Ina168 (Yoshida et al., 2009 [13]) and AvrPii-C-FL_F/R was based on AvrPii-C specific sequence created by Tail-PCR approach. Isolate entries 1 to 10 were as CHL22, CHL611, CHL624, CHL625, EHL0354, CHL346, CHL584, CHL590, EHL0445, EHL0624; M, DNA ladder (2000 bp). The phenotypes were determined when challenged with isolates on the *Pii* carrier, IRBLi-F5 (A, avirulent). (**C**) Functionality and specificity of transgenic isolates derived from both haplotypes as well as their donor and recipient isolates those were challenged on five monogenic lines carrying individual resistance genes.

All the ten avirulent isolates selected from various Chinese *Mo* populations were amplified the expected CDS products with the key primer pair, AvrPii-J/C-CDS_F/R, whereas only the first five isolates were with the expected FL amplicons by the key primer pair, AvrPii-J-FL_F/R, indicating that sequences of the second primer pair might be less matched with those of the second five isolates (Figure 1A,B). The key primer pair, AvrPii-C-FL_F/R, which was devised based on the specific sequences generated from both 5′ and 3′ regions of the representative isolates CHL346 via Tail-PCR system (Figure S1), was employed to re-amplify the ten isolates. The reverse result was observed, i.e., the first five isolates were without any amplicon, except for #1, CHL22, and the second five one with the expected products. The reasonable explanation for genotype of the exceptional isolate CHL22 is that it carries two copies of *AvrPii*, i.e., *AvrPii*-J and *AvrPii*-C, which resembles some isolates shown in Table S2.

Thus, the key primer pairs, AvrPii-J-RS_F/R, and AvrPii-C-RS_F/R, respectively, were used for amplifying the first and second five isolates for the haplotypic amplicon-based sequencing. As expected, there were extensive variations in both 5′ and 3′ regions, and intermediate ones in CDS region between the two haplotypes (two typical sequence difference as shown in Figures S1 and S2). The target gene from the first five isolates was temporally defined as *AvrPii*-J, and that from the second five isolates as *AvrPii*-C. Transgenic progenies derived from both haplotypes expressed a consistent functionality and specificity on five monogenic rice lines carrying *Pii* and other four individual resistance genes (Figure 1C). Noticeably, both haplotypes expressed moderately avirulent to the *Pii* carrier. 

Altogether, the new haplotype, *AvrPii*-C (GenBank MK830673), is a novel and functional one in the *AvrPii* family.

### 3.2. Performance of Haplotype-Chimeric Mutant

**Figure 2:**
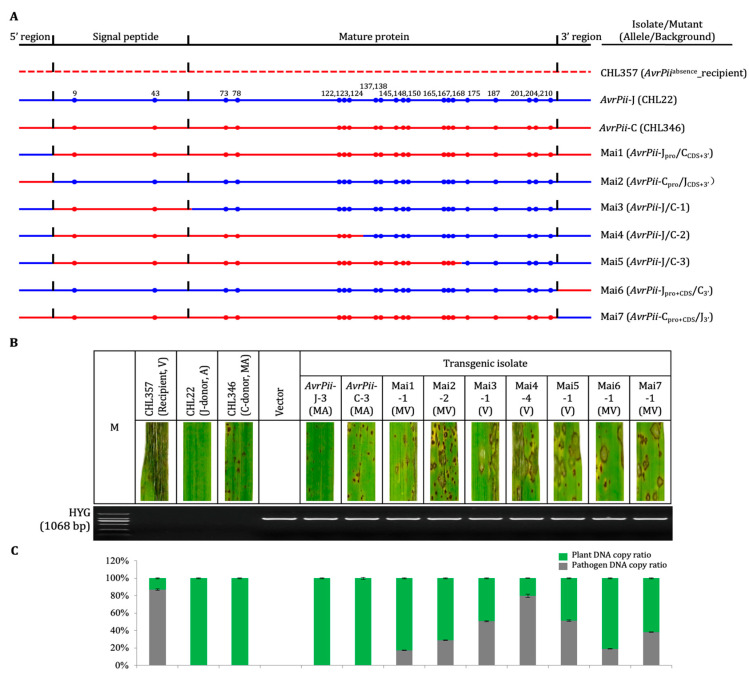
Validation of functions of haplotype-chimeric mutants of *AvrPii*. (**A**) Genomic schemas of target genes of the two haplotypes and their seven mutants, plus the recipient isolate, CHL357. The blue represents *AvrPii*-J and red *AvrPii*-C; absence is shown by dotted lines. The sequences have not been drawn to scale. (**B**) Phenotypes and genotypes of both haplotypes and seven mutants plus the two donor and one recipient isolate, the former one was determined by inoculation with transgenic isolates as well as their donor and recipient isolates on the *Pii* carrier, the latter one by genotyping with the vector-embedded selective marker (HYG). A, Avirulent; V, virulent; MA, moderately avirulent; MV, moderately virulent; M, DNA ladder (2000 bp); (**C**) Quantification of Plant and fungal DNA extracted from representative regions of leaves selected from the above transgenic isolates via qPCR approach. The plant and fungal DNA, respectively, were inferred from the abundance of the rice *Ubi* and the *Mo Pot2* amplicons; the data shown was given in the form mean ± standard deviation (*n* = 3). Similar results were obtained from two biological replicates each with three technical repeats.

Compared to moderately avirulent performance of transgenic isolates derived from both wild haplotypes, reactions of each two haplotype-chimeric mutants derived from both 5′ (Mai1-2) and 3′ (Mai6-7) were quantified as moderately virulent, compared to the moderate avirulent symptoms caused by both wild types, where no fungal growth could be detected by qPCR (Figure 2). It suggests that both 5′ and 3′ regions were specific to the individual wild haplotypes because replacement of either 5′ or 3′ regions led to decline expression of their avirulence against the *Pii* carrier (Figure S3; Table S3), while the performance of three haplotype-chimeric mutants, which were derived from CDS regions (Mai3-5), were almost changed from moderately avirulent to virulent. It indicates that the integrity of CDS was crucial to express functionality of the individual haplotypes. Correspondingly, the protein structures of CDS-derived chimeric mutants were distinct from both wild type ones, i.e., Mai1-2 and 6-7 (Figure S4). 

Furthermore, there was not any positive interaction in Bait/Prey reciprocal combinations between four mature proteins (AvrPii-J^WT^_20-70_, AvrPii-J/C-2_20-70_, AvrPii-J/C-3_20-70_, and AvrPii-C^WT^_20-70_) and a series of domains of the paired proteins of Pii (Figure S5A,B). The results were consistent with those reported in the previous studies [3,26]. There were three positive reactions only in Prey/Bait combinations between five full-length proteins AvrPii (Prey) and a series of domains of the paired proteins of Pii (Bait), i.e., a weaker interaction between AvrPii-J/C-3_FL_ and Pii-1_CC_, and a stronger and weaker interactions, respectively, between AvrPii-C^WT^_FL_ and Pii-1_CC_ and Pii-2_LRR_ (Figure S5C,D). These results indicate that the younger prey of the AvrPii-C, particularly the interval from #131 to #213 (Figure 2; Table S3), could be interacted with the cognate bait of the Pii, during their long-term co-evolution process. 

### 3.3. Population Structure of the AvrPii Family

**Figure 3:**
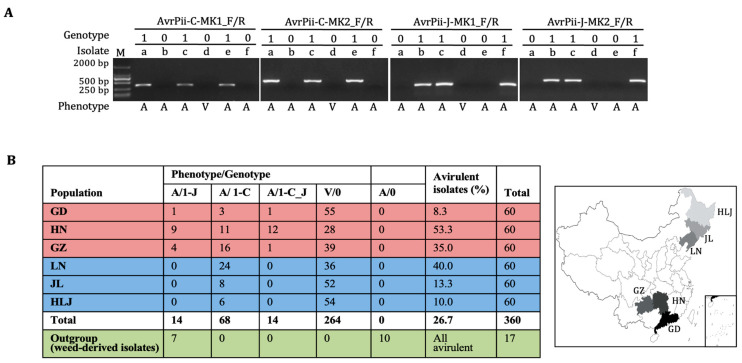
Population structures of *AvrPii* in the six *M*. *oryzae* populations collected in China. (**A**) Genotyping of the six typical *Mo* isolates. The two codes, 1 (presence of amplicon) and 0 (absence of amplicon), were used for genotyping. The phenotype of each isolate when challenged on the *Pii* carrier was either A (avirulent) or V (virulent). Because MK1- and MK2-based genotypes were coincident, the integrated phenotype/genotype is simply given to each isolate as: a, EHL0314 (A/1-C); b, EHL0317 (A/1-J); c, EHL0319 (A/1-C_J); d, EHL0313 (V/0); e, CHL2458 (A/1-C); f, CHL611 (A/1-J); M1, DNA ladder (2000 bp). (**B**) The distribution of combinations of phenotypes/genotypes among the six *Mo* populations. The weed-derived isolates were assorted as an outgroup. GD, Guangdong, HN, Hunan, GZ, Guizhou, LN, Liaoning, JL, Jilin, and HLJ, Heilongjiang (also see Supplementary Table S2).

As for *Pii*-based phenotyping, frequencies of avirulent isolates ranged from 8.3% (GD) to 53.3% (HN), indicating that the *AvrPii* family was less avirulent and diverse in both southern and northern regions in China (Figure 3; Table S2). As for the two haplotype-specific marker-based genotyping, the results derived from the shorter fragment markers (MK1) were completely repeated by that from the longer one (MK2), thereby simply giving only four genotypes, 0, 1-C, 1-J, and 1-C_J, among six *Mo* populations derived from rice (Figure 1A and Figure 3A; Table S2). Intriguingly, combinations of phenotypes/genotypes were also only four, V/0, A/1-C, A/1-J, and A/1-C_J, because all the *Pii*-virulent isolates were restricted to genotype 0, whereas the *Pii*-avirulent ones were with the remaining three. Furthermore, all the four combinations were detected in the three southern populations, and only two A/1-C and V/0 in all the northern ones, suggesting that genic diversity in the southern region was higher than those in the northern one (Figure 3B). Together, the *AvrPii* family was rarer and less diverse in all the six *Mo* populations, particularly in the northern region.

Regarding outgroup, all 17 weed-derived isolates could not infect rice, and only seven were identified as A/1-J, indicating that the *AvrPii*-J, which emerged before rice domestication, is older than *AvrPii*-C (Figure 3B; Table S2). 

### 3.4. Evolutionary and Regional Divergence of the AvrPii Family

The genetic diversity of the *AvrPii* family among the six populations was explored in some detail by re-sequencing all 110 amplicons derived from 96 isolates, of which 14 carry both haplotypes (Figure 3B; Table S2). The CDS harbored 20 SNPs, of which eleven were non-synonymous. The non-signal domain harbored 18 of the SNP sites, while the other two were within the signal peptide domain; as a result, the level of nucleotide diversity in the former domain (0.045) was higher than in the latter (0.013) (Table 1). As to the individual haplotypes, only one segregating site was found in *AvrPii*-C group and none in *AvrPii*-J one, indicating that there were extremely less diverse in both haplotypes. Regarding the regional groups, both haplotypes were identified in the southern group, thereby raising 20 segregating sites for reaching a higher level (0.045) of the *AvrPii* diversity. On the contrary, only one haplotype, *AvrPii*-C, was placed in the northern one without any segregating site. Again, it was clearly suggested that diversity of the *AvrPii* in the southern group was largely higher than that in the northern one. 

In all *AvrPii* group, the first set of selection-related parameters D* and F* in both entire (D* = 1.74; F* = 2.65) and non-signal peptide region (D* = 1.68; F* = 2.59) were positive and higher than those in signal peptide region (D* = 0.68; F* = 1.11), indicating that the target locus has been experienced a stronger balancing selection in both regions and a slighter one in signal peptide region (Table 1). In the haplotype groups, only slight balancing selection pressure (D* = 0.51; F* = 0.79) was detected within the haplotype *AvrPii*-C group, and none in the haplotype *AvrPii*-J one. As to the regional group, there has been a stronger balancing selection pressure (D* = 1.72; F* = 3.04) on the *AvrPii* family in the southern group, but none in the northern one. As to the second set of selection-related parameters (*Ka*, *Ks*, and *Ka*/*Ks*), *Ka*/*Ks* in both all *AvrPii* and the southern groups were less than 0.57, indicating that the *AvrPii* family has been imposed by a stronger purifying selection (negative selection) in both groups. While a slight positive selection (*Ka* > *Ks*) was detected within the haplotype *AvrPii*-C group. Intriguingly, neither positive nor balancing selection pressures was detected in haplotype *AvrPii*-J, the regional north, and out groups (Table 1). 

## 4. Discussion

### 4.1. The Lesson Learned from Case Study on Population Structures of the AvrPii Family

Having identified the novel haplotype, *AvrPii*-C, dynamic population structures of the *AvrPii* family were finely dissected among six *Mo* populations, each three from southern and northern regions of China (Figure 1, Figure 2 and Figure 3). Whereas the apparent contradiction between the higher detected number of avirulent isolates and the lower number of detected avirulence genes of *AvrPii* were observed in various *Mo* populations previously [3,11,14,15], compared to the lower rate of presence of *AvrPii* being detected, the higher rate of *Pii*-avirulent isolates was partly due to *Pii* independent that was conveyed by another gene pair of *Avr*/*R* gene in such combinations [3]. It was, however, certainly conceivable that failing in detecting a yet unknown haplotype *AvrPii*-C in such *Pii*-avirulent isolates as reported in the current study was the major reason for creating the numerical gaps and/or the lower rates of presence of *AvrPii*. When revisiting all PCR primer sequences used in the previous studies [3,11,14,15], it was revealed that all such primers were designed from the known reference sequence of the *AvrPii*-J (AB498874 that resembles to CHL22 as shown in Figure S1) [2], those were not matching enough for amplifying the *AvrPii*-C as shown in Figure 1. Because the proportion of the *AvrPii*-C was almost five times higher than that of the *AvrPii*-J in the six *Mo* populations tested in the present research (Figure 3), in addition to the fact that higher resistance frequencies of the *Pii* carrier IRBLi-F5 were detected in Japan [27], Bangladesh [28], Kenya [29], and Vietnam [30], it was conceivable that the numerical gaps and/or extremely lower proportions of presence of *AvrPii* in various populations were largely due to missing the novel haplotype, *AvrPii*-C [3,11,14,15]. 

Considering haplotype divergence is one of the most important mechanisms underpinning *Avr* gene evolution in various plant pathosystems [3,7,15,31,32,33,34,35,36,37,38], the lesson learned from case studies on population structures of the *AvrPii* family is that much attention should be focused on haplotype divergence of the target gene when there were certain numerical gaps or extremely lower proportions of presence of the target gene as reported in the previous investigations [3,11,14,15]. Briefly, the population structure of individual target gene(s) is better dissected by both phenotypic and genotypic datasets so that the reasons for the numerical gap and/or extremely lower proportion of target gene could be easily found. If done, the specific sequence for unknown haplotype, which is distinct from the known one, should be created via the Tail-PCR system in a case of fewer-than-enough reference sequences available, and/or through gene similarity analysis using reference sequence of the known one for inquiring whether there was/were candidate distinct haplotype(s) in public database (as shown in Figures S1 and S2). Then primer sets for the novel haplotype, if any, should be re-devised based the haplotype-specific sequence for genotyping and resequencing (as shown in Figure 1) [3]. Furthermore, the novel haplotype(s) might be functionally characterized via transformation test, if possible and necessary. 

### 4.2. The Lesson Learned from Evolutionary Story of the AvrPii Family

Accumulating evidence from population genetic study on various *Avr* genes demonstrated that the gene-for-gene principle has played a pivotal role in featuring their population structures [3,7,8,9,11,34,39,40]. As per the definitions of three kinds of the key selections, stronger balancing and purifying selection forces were exerted on the Chinese populations and only positive selection pressure was exerted on the Thai population, when datasets available in both Lu et al., 2019 [3] and Sirisathaworn et al., 2017 [11] were re-assayed by the same approaches employed in the current study (Table S4). As pathogen *Avr* genes have generally evolved in response to positive selection [7,8,9,11,39,40,41], the unique population structures of the *AvrPii* family found in China have significant implications for understanding how the *AvrPii* family has kept an artful balance and purity among its members (haplotypes), those keenly interact with the cognate *R* gene, *Pii*, under gene-for-gene relationships. 

Furthermore, several intriguing points, which were extracted from five comparable groups in the current study, should be marked. First, it could be clearly deemed that the haplotype *AvrPii*-C was a mutant emerged after rice domestication, and the haplotype *AvrPii*-J as wild type has emerged before rice domestication (Figure 3; Table S2). Second, the population structures between haplotypes *AvrPii*-C and *AvrPii*-J groups were slightly different, the former one received weaker balancing and positive selection forces, thereby scattering in all the six *Mo* populations across China, and the latter one has received a weaker purifying and even neutral selection pressure, thereby being maintained and restricted in the three southern populations (Table 1; Figure 3). Third, the population structures between southern and northern groups were distinct. The former one has experienced both stronger balancing and purifying selection pressure, thereby keeping all the four combinations of phenotypes/genotypes (A/1-J, A/1-C, A/1-C_J, and V/0), and the latter one has received a weaker purifying and even neutral selection pressure, thereby keeping maintained only two (A/1-C and V/0), which led to show certainly higher genetic diversity in the southern region. Fourth, higher frequencies of avirulent isolates were detected in southern HN (53.3%) and GZ (35.0%) and in northern LN (40.0%), but over three-fold lower ones in their neighboring regions, i.e., the southern GD (8.3%), and the northern JL (13.3%) and HLJ (10.0%) (Figure 3). That, in turn, indicated that the cognate *R* gene, *Pii*, could be continuously used as basic and critical resistance resource in HN, GZ, and LN populations, even though it was recognized as one of the extremely rarer and weaker *R* genes in the Chinese rice breeding programs [2,5,12,42,43,44]. Again, it provided further weight to the idea that the *AvrPii* family was a smarter one to keep a sophisticated balance and purity not only its members but also its living places. 

It is worthy learning more marvelous lessons from evolution stories of the *AvrPii* family featured by its balanced, purified, and directed haplotypes in global *Mo* populations including trans-species ones. 

## 5. Conclusions

The study has identified a sequence-distinct and functional haplotype *AvrPii*-C, compared to the known one, *AvrPii*-J. It was believed that failing in amplifying of *AvrPii*-C was the major reason for causing the numerical gaps between higher proportions of the *Pii*-avirulent isolates and/or extremely lower proportions of presence of *AvrPii* in various populations previously reported. The unique population structure of the *AvrPii* family was shaped by its balanced, purified, and directed haplotypes in the Chinese populations. The *AvrPii*-C was recognized as a younger one that emerged after rice domestication. The cognate resistance gene *Pii* could be continuously used as a basic and critical resistance resource in Hunan, Guizhou, and Liaoning provinces, China. 

## 6. Patent

Qinghua Pan, Xing Wang, Cheng Li, Jianqiang Wen, Yu Zhang, Ling Wang. Identification and applications of the avirulence gene *AvrPii*-C of *Magnaporthe oryzae* (ZL201811273480.3, Granted on 14 August 2020)

## Figures and Tables

**Table 1:** Evolutionary parameters of coding sequences of the *AvrPii* family of *M*. *oryzae.*

Comparable Group ^a^	*S* ^b^	*π* ^c^	D* ^d^	F*	*Ka* ^e^	*Ks* ^f^	*Ka* */Ks*
**All *AvrPii*** (∑110)							
Entire coding region	20	0.037	1.74 ^β^	2.65 ^β^	193.8	357.6	0.54
Non-signal peptide region	18	0.045	1.68 ^α^	2.59 ^β^	246.8	434.8	0.57
Signal peptide region	2	0.013	0.68	1.11	54.9	166.0	0.33
**Haplotype *AvrPii*-J** (∑28)							
Entire coding region	0	0.000	0.00	0.00	0.0	0.0	*Ka* = *Ks*
Non-signal peptide region	0	0.000	0.00	0.00	0.0	0.0	*Ka* = *Ks*
Signal peptide region	0	0.000	0.00	0.00	0.0	0.0	*Ka* = *Ks*
**Haplotype *AvrPii*-C** (∑82)							
Entire coding region	1	0.002	0.51	0.79	8.1	0.0	*Ka* > *Ks*
Non-signal peptide region	1	0.002	0.51	0.79	11.0	0.0	*Ka* > *Ks*
Signal peptide region	0	0.000	0.00	0.00	0.0	0.0	*Ka* = *Ks*
**Regional group** (∑110)							
South (*AvrPii*-J, -C; ∑72)	20	0.045	1.72 ^β^	3.04 ^β^	100.8	192.2	0.52
North (*AvrPii*-C; ∑38)	0	0.000	0.00	0.00	0.0	0.0	*Ka* = *Ks*
**Outgroup** (∑7)							
Weed-derived isolates (*AvrPii*-J)	0	0.000	0.00	0.00	0.0	0.0	*Ka* = *Ks*

^a^ Total number of each group was according to the results shown in Figure 3, particularly, 14 isolates carried both *AvrPii*-C and *AvrPii*-J, those were courted out two times (also see details in Table S2). ^b^ Number of segregating sites. ^c^ Nei’s nucleotide diversity based on silent site. ^d^ Fu and Li’s D* and F*, and ^α^, ^β^ represents statistical significance at *p* < 0.05, 0.02 level, respectively. ^e^ Nonsynonymous. ^f^ Synonymous.

## Data Availability

Not applicable.

## Supplementary Materials

The following supporting information can be downloaded at: https://www.mdpi.com/article/10.3390/genes14051065/s1, Figure S1: Discovery of a novel haplotype *AvrPii*-C in Chinese populations of *M*. *oryzae*; Figure S2: Alignment of the full-length sequences of both haplotypes, *AvrPii*-J and *AvrPii*-C; Figure S3: Constructure of haplotype-chimeric mutants by PCR-based domain swapping from *AvrPii-*J to *AvrPii-*C; Figure S4: The 3D-structures of a set of five AvrPii proteins; Figure S5: Interactions between AvrPii and Pii proteins in the Y2H system; Table S1. PCR primers and programs used in the current study; Table S2. Genotypes and phenotypes of 377 isolates of *M. oryzae* characterized in the current study [45]; Table S3. The full-length sequences of seven haplotype-chimeric mutants of the *AvrPii* family; Table S4. Comparison of evolutionary parameters of the *AvrPii* family among three *Mo* populations, of which parameters of the two populations previously reported were re-computed by the same approaches used in the current study.
