# Supplementary material for: The Lesson Learned from the Unique Evolutionary Story of Avirulence Gene AvrPii of Magnaporthe oryzae
Source: Genes (Basel). 2023 May 11;14(5):1065. doi: 10.3390/genes14051065 (PMC10218241; doi:10.3390/genes14051065)
Supplement: Supplementary file 1 [file genes-14-01065-s001.zip › genes-2373462-supplementary/23-5-7 Supplementary Materials for AvrPii/Figure S1. Tail- PCR of AvrPii-C.pptx]

## Slide 1
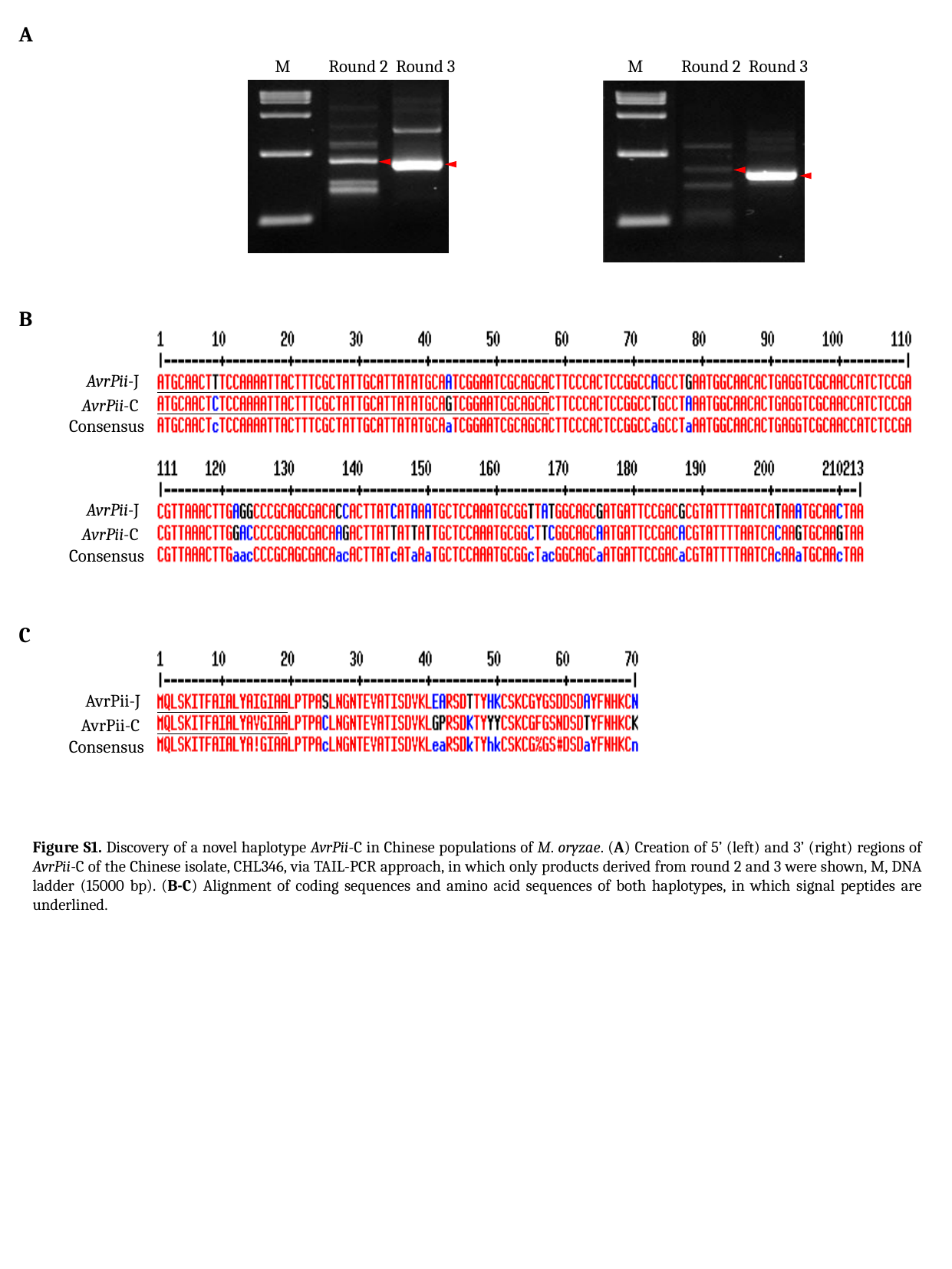

A
M
Round 2
Round 3
M
Round 2
Round 3
B
AvrPii-J
AvrPii-C
Consensus
AvrPii-J
AvrPii-C
Consensus
C
AvrPii-J
AvrPii-C
Consensus
Figure S1. Discovery of a novel haplotype AvrPii-C in Chinese populations of M. oryzae. (A) Creation of 5’ (left) and 3’ (right) regions of AvrPii-C of the Chinese isolate, CHL346, via TAIL-PCR approach, in which only products derived from round 2 and 3 were shown, M, DNA ladder (15000 bp). (B-C) Alignment of coding sequences and amino acid sequences of both haplotypes, in which signal peptides are underlined.
