# Supplementary material for: The Lesson Learned from the Unique Evolutionary Story of Avirulence Gene AvrPii of Magnaporthe oryzae
Source: Genes (Basel). 2023 May 11;14(5):1065. doi: 10.3390/genes14051065 (PMC10218241; doi:10.3390/genes14051065)
Supplement: Supplementary file 1 [file genes-14-01065-s001.zip › genes-2373462-supplementary/23-5-7 Supplementary Materials for AvrPii/Figure S2. Divergence and primers of AvrPii-C_J.pptx]

## Slide 1
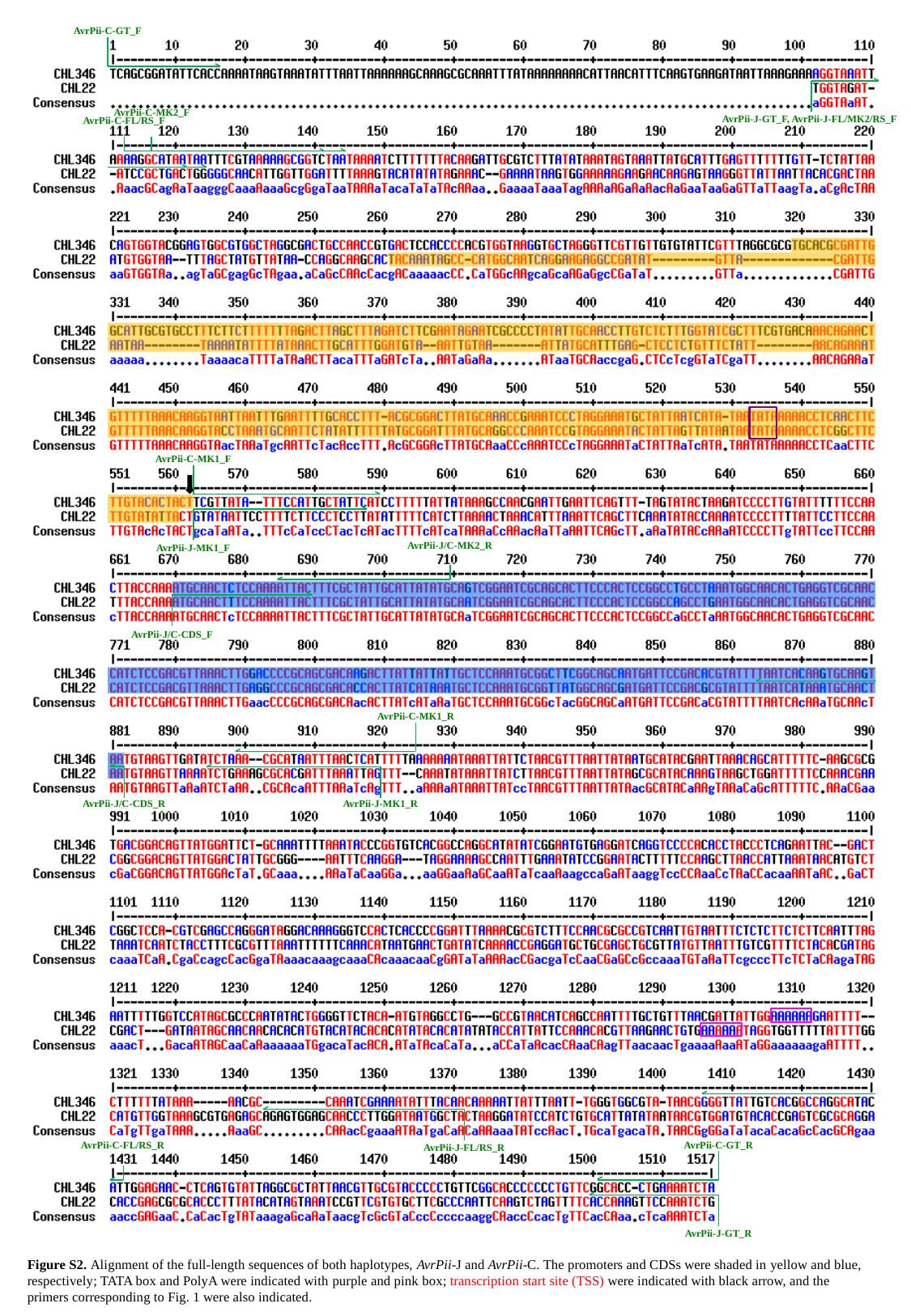

AvrPii-C-GT_F
AvrPii-C-MK2_F
AvrPii-J-GT_F, AvrPii-J-FL/MK2/RS_F
AvrPii-C-FL/RS_F
AvrPii-C-MK1_F
AvrPii-J/C-MK2_R
AvrPii-J-MK1_F
AvrPii-J/C-CDS_F
AvrPii-C-MK1_R
AvrPii-J/C-CDS_R
AvrPii-J-MK1_R
AvrPii-C-FL/RS_R
AvrPii-C-GT_R
AvrPii-J-FL/RS_R
AvrPii-J-GT_R
Figure S2. Alignment of the full-length sequences of both haplotypes, AvrPii-J and AvrPii-C. The promoters and CDSs were shaded in yellow and blue, respectively; TATA box and PolyA were indicated with purple and pink box; transcription start site (TSS) were indicated with black arrow, and the primers corresponding to Fig. 1 were also indicated.
