# Supplementary material for: The Lesson Learned from the Unique Evolutionary Story of Avirulence Gene AvrPii of Magnaporthe oryzae
Source: Genes (Basel). 2023 May 11;14(5):1065. doi: 10.3390/genes14051065 (PMC10218241; doi:10.3390/genes14051065)
Supplement: Supplementary file 1 [file genes-14-01065-s001.zip › genes-2373462-supplementary/23-5-7 Supplementary Materials for AvrPii/Figure S3. AvrPii-C_J domain-swapping.pptx]

## Slide 1
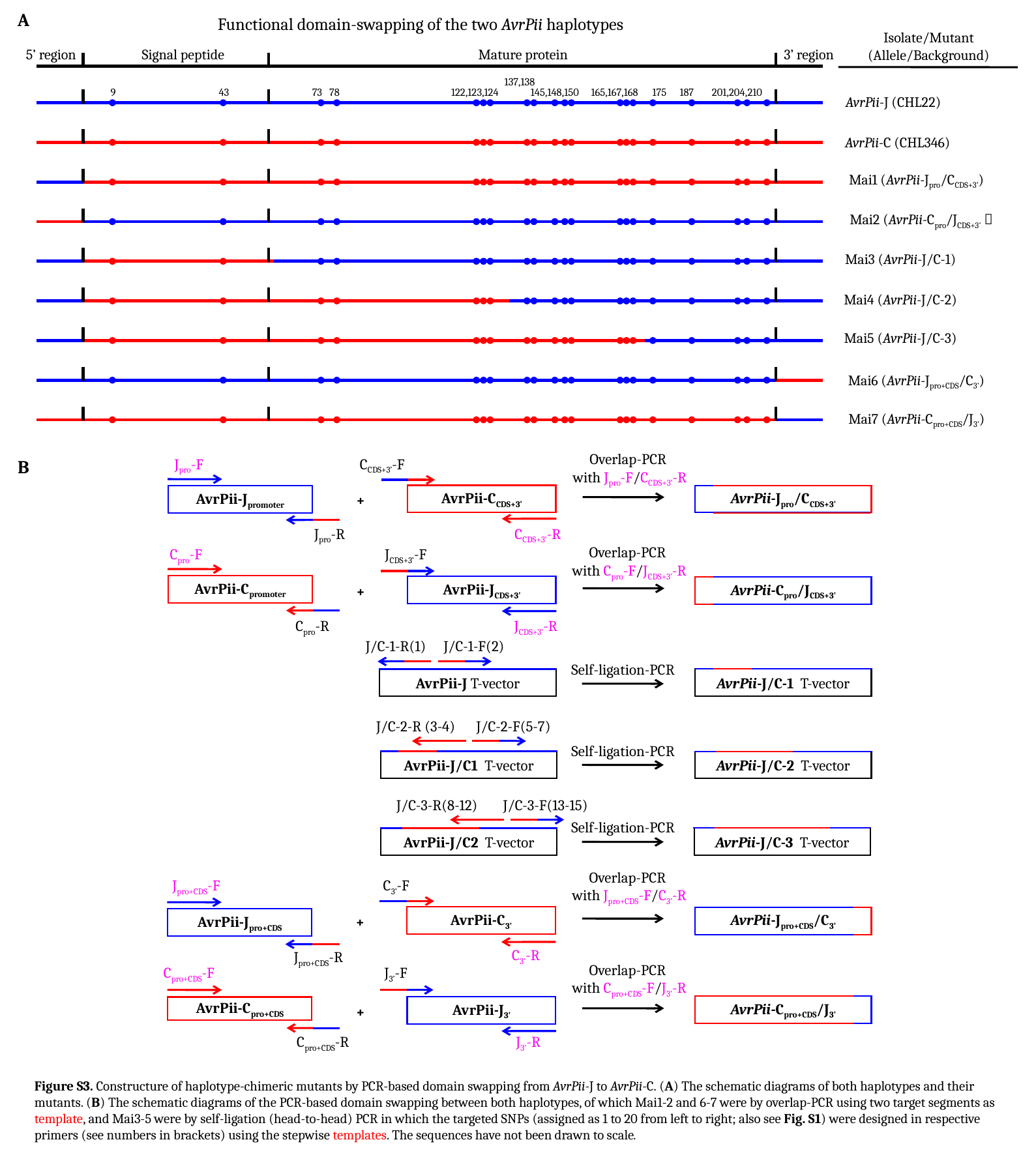

Functional domain-swapping of the two AvrPii haplotypes
5’ region
Signal peptide
Mature protein
3’ region
137,138
9
43
73
78
122,123,124
145,148,150
165,167,168
175
187
201,204,210
Isolate/Mutant
(Allele/Background)
AvrPii-J (CHL22)
AvrPii-C (CHL346)
Mai1 (AvrPii-Jpro/CCDS+3’)
Mai2 (AvrPii-Cpro/JCDS+3’）
Mai3 (AvrPii-J/C-1)
Mai4 (AvrPii-J/C-2)
Mai5 (AvrPii-J/C-3)
Mai6 (AvrPii-Jpro+CDS/C3’)
Mai7 (AvrPii-Cpro+CDS/J3’)
A
Jpro-F
CCDS+3’-F
Overlap-PCR
with Jpro-F/CCDS+3’-R
AvrPii-Jpro/CCDS+3’
AvrPii-CCDS+3’
AvrPii-Jpromoter
+
Jpro-R
CCDS+3’-R
B
Overlap-PCR
with Cpro-F/JCDS+3’-R
Cpro-F
JCDS+3’-F
AvrPii-Cpromoter
AvrPii-Cpro/JCDS+3’
AvrPii-JCDS+3’
+
Cpro-R
JCDS+3’-R
J/C-1-R(1)
J/C-1-F(2)
Self-ligation-PCR
AvrPii-J T-vector
AvrPii-J/C-1 T-vector
J/C-2-R (3-4)
J/C-2-F(5-7)
Self-ligation-PCR
AvrPii-J/C1 T-vector
AvrPii-J/C-2 T-vector
J/C-3-R(8-12)
J/C-3-F(13-15)
Self-ligation-PCR
AvrPii-J/C2 T-vector
AvrPii-J/C-3 T-vector
Overlap-PCR
with Jpro+CDS-F/C3’-R
Jpro+CDS-F
C3’-F
AvrPii-Jpro+CDS/C3’
AvrPii-C3’
+
AvrPii-Jpro+CDS
C3’-R
Jpro+CDS-R
Overlap-PCR
with Cpro+CDS-F/J3’-R
Cpro+CDS-F
J3’-F
AvrPii-Cpro+CDS/J3’
AvrPii-J3’
AvrPii-Cpro+CDS
+
Cpro+CDS-R
J3’-R
Figure S3. Constructure of haplotype-chimeric mutants by PCR-based domain swapping from AvrPii-J to AvrPii-C. (A) The schematic diagrams of both haplotypes and their mutants. (B) The schematic diagrams of the PCR-based domain swapping between both haplotypes, of which Mai1-2 and 6-7 were by overlap-PCR using two target segments as template, and Mai3-5 were by self-ligation (head-to-head) PCR in which the targeted SNPs (assigned as 1 to 20 from left to right; also see Fig. S1) were designed in respective primers (see numbers in brackets) using the stepwise templates. The sequences have not been drawn to scale.
