# Supplementary material for: The Lesson Learned from the Unique Evolutionary Story of Avirulence Gene AvrPii of Magnaporthe oryzae
Source: Genes (Basel). 2023 May 11;14(5):1065. doi: 10.3390/genes14051065 (PMC10218241; doi:10.3390/genes14051065)
Supplement: Supplementary file 1 [file genes-14-01065-s001.zip › genes-2373462-supplementary/23-5-7 Supplementary Materials for AvrPii/Figure S4. AvrPii mutants_3D.pptx]

## Slide 1
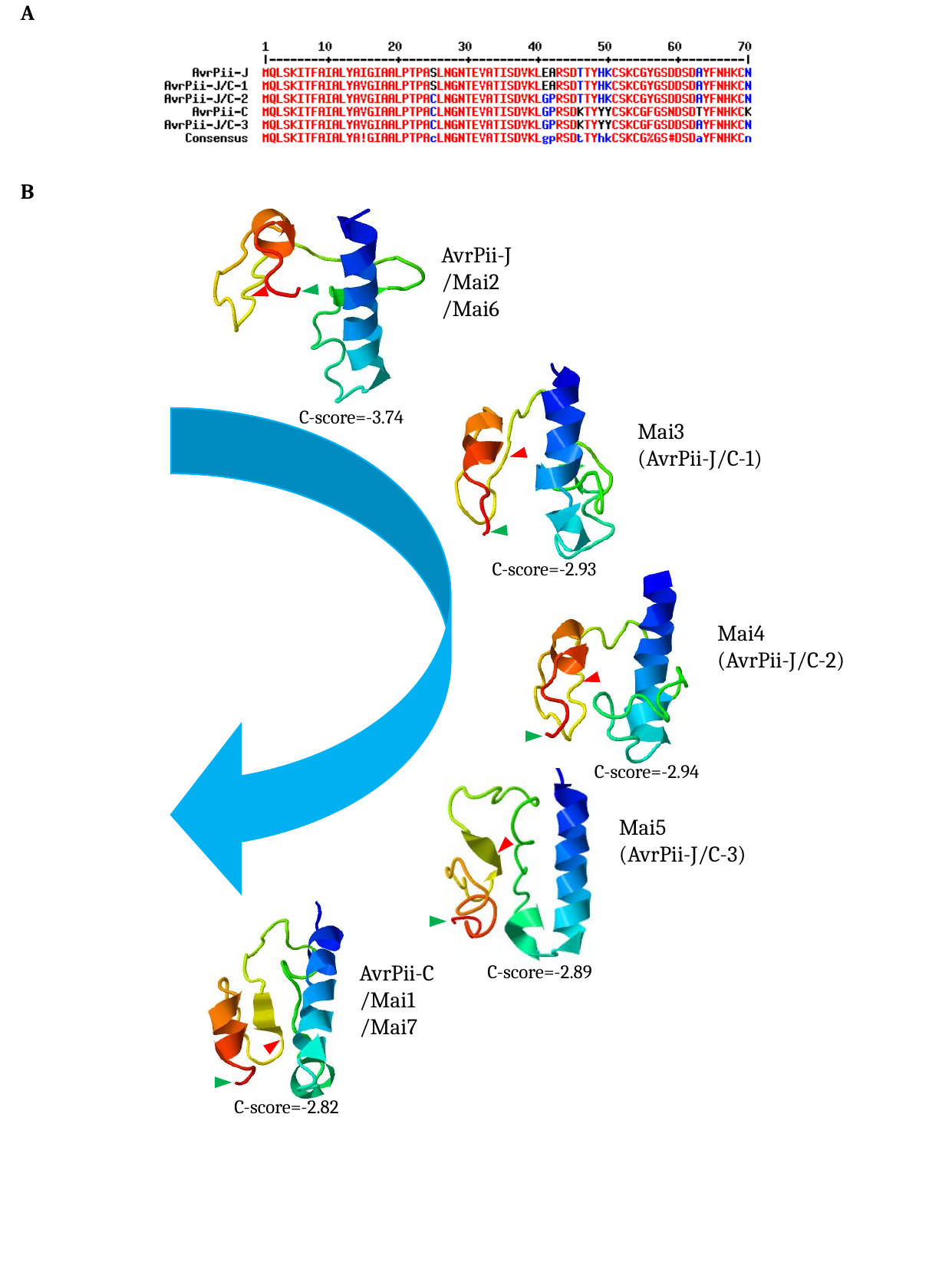

A
B
AvrPii-J
/Mai2
/Mai6
C-score=-3.74
Mai3
(AvrPii-J/C-1)
C-score=-2.93
Mai4
(AvrPii-J/C-2)
C-score=-2.94
Mai5
(AvrPii-J/C-3)
AvrPii-C
/Mai1
/Mai7
C-score=-2.89
C-score=-2.82

## Slide 2
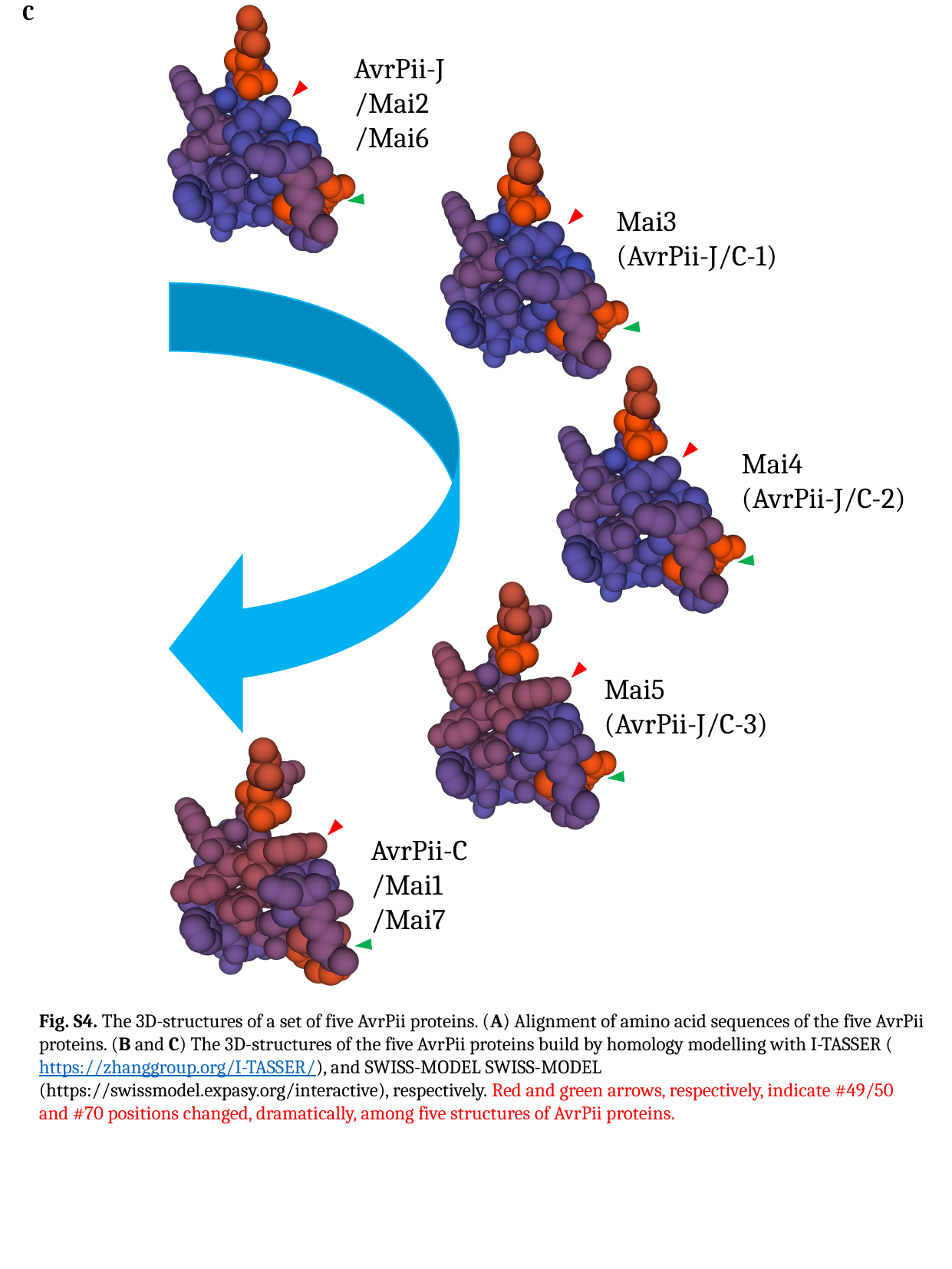

C
AvrPii-J
/Mai2
/Mai6
Mai3
(AvrPii-J/C-1)
Mai4
(AvrPii-J/C-2)
Mai5
(AvrPii-J/C-3)
AvrPii-C
/Mai1
/Mai7
Fig. S4. The 3D-structures of a set of five AvrPii proteins. (A) Alignment of amino acid sequences of the five AvrPii proteins. (B and C) The 3D-structures of the five AvrPii proteins build by homology modelling with I-TASSER (https://zhanggroup.org/I-TASSER/), and SWISS-MODEL SWISS-MODEL (https://swissmodel.expasy.org/interactive), respectively. Red and green arrows, respectively, indicate #49/50 and #70 positions changed, dramatically, among five structures of AvrPii proteins.
