# Supplementary material for: The Lesson Learned from the Unique Evolutionary Story of Avirulence Gene AvrPii of Magnaporthe oryzae
Source: Genes (Basel). 2023 May 11;14(5):1065. doi: 10.3390/genes14051065 (PMC10218241; doi:10.3390/genes14051065)
Supplement: Supplementary file 1 [file genes-14-01065-s001.zip › genes-2373462-supplementary/23-5-7 Supplementary Materials for AvrPii/Figure S5. AvrPii_Pii Y2H.pptx]

## Slide 1
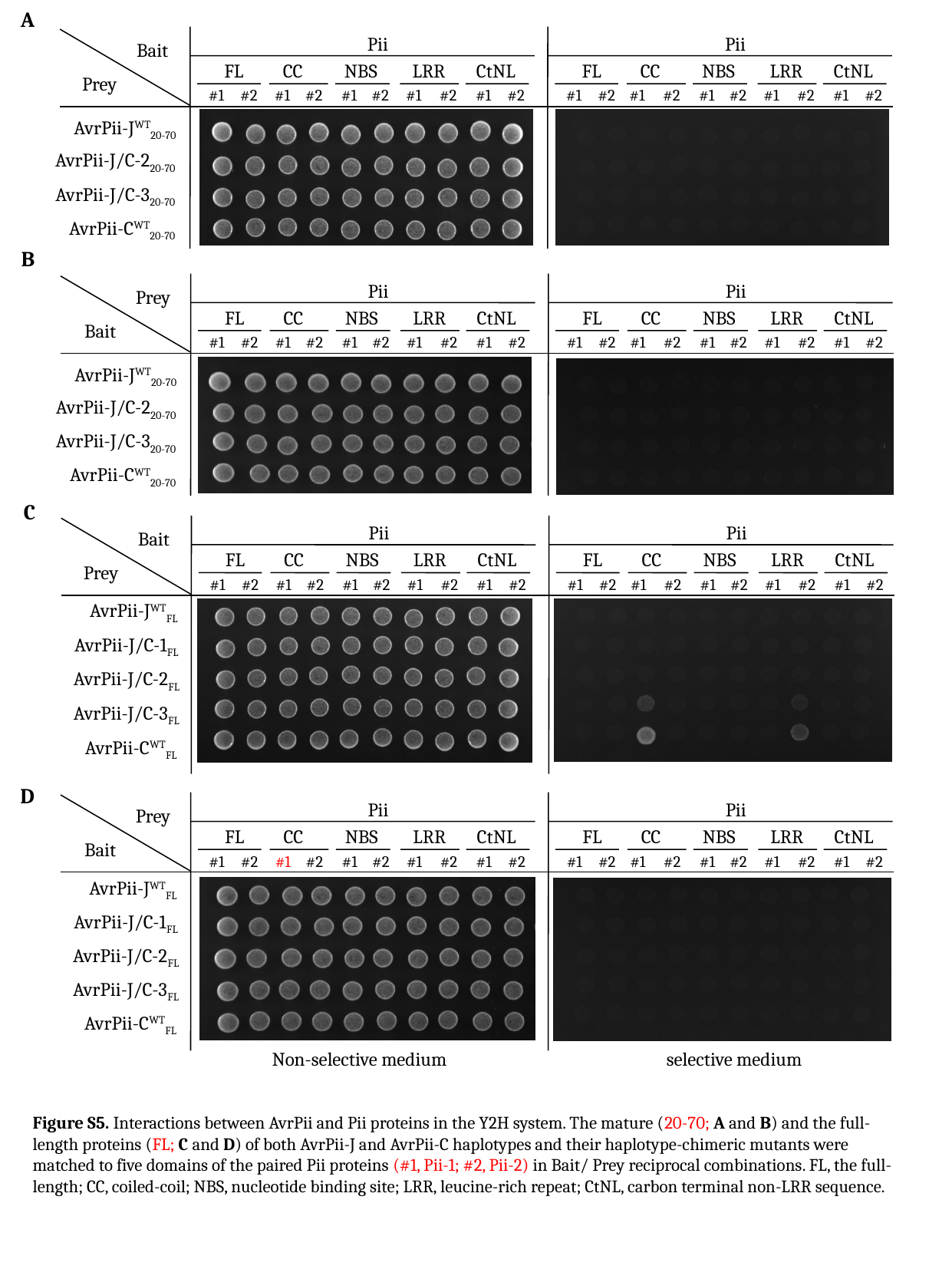

A
Pii
Pii
Bait
FL
CC
NBS
LRR
CtNL
FL
CC
NBS
LRR
CtNL
Prey
#1
#2
#1
#2
#1
#2
#1
#2
#1
#2
#1
#2
#1
#2
#1
#2
#1
#2
#1
#2
AvrPii-JWT20-70
AvrPii-J/C-220-70
AvrPii-J/C-320-70
AvrPii-CWT20-70
B
Pii
Pii
Prey
FL
CC
NBS
LRR
CtNL
FL
CC
NBS
LRR
CtNL
Bait
#1
#2
#1
#2
#1
#2
#1
#2
#1
#2
#1
#2
#1
#2
#1
#2
#1
#2
#1
#2
AvrPii-JWT20-70
AvrPii-J/C-220-70
AvrPii-J/C-320-70
AvrPii-CWT20-70
C
Pii
Pii
Bait
FL
CC
NBS
LRR
CtNL
FL
CC
NBS
LRR
CtNL
Prey
#1
#2
#1
#2
#1
#2
#1
#2
#1
#2
#1
#2
#1
#2
#1
#2
#1
#2
#1
#2
AvrPii-JWTFL
AvrPii-J/C-1FL
AvrPii-J/C-2FL
AvrPii-J/C-3FL
AvrPii-CWTFL
D
Pii
Pii
Prey
FL
CC
NBS
LRR
CtNL
FL
CC
NBS
LRR
CtNL
Bait
#1
#2
#1
#2
#1
#2
#1
#2
#1
#2
#1
#2
#1
#2
#1
#2
#1
#2
#1
#2
AvrPii-JWTFL
AvrPii-J/C-1FL
AvrPii-J/C-2FL
AvrPii-J/C-3FL
AvrPii-CWTFL
Non-selective medium
selective medium
Figure S5. Interactions between AvrPii and Pii proteins in the Y2H system. The mature (20-70; A and B) and the full-length proteins (FL; C and D) of both AvrPii-J and AvrPii-C haplotypes and their haplotype-chimeric mutants were matched to five domains of the paired Pii proteins (#1, Pii-1; #2, Pii-2) in Bait/ Prey reciprocal combinations. FL, the full-length; CC, coiled-coil; NBS, nucleotide binding site; LRR, leucine-rich repeat; CtNL, carbon terminal non-LRR sequence.
