# Supplementary material for: The Lesson Learned from the Unique Evolutionary Story of Avirulence Gene AvrPii of Magnaporthe oryzae
Source: Genes (Basel). 2023 May 11;14(5):1065. doi: 10.3390/genes14051065 (PMC10218241; doi:10.3390/genes14051065)
Supplement: Supplementary file 1 [file genes-14-01065-s001.zip › genes-2373462-supplementary/23-5-7 Supplementary Materials for AvrPii/Table S1. Primers & PCR programs.pdf]

Table S1. PCR primers and programs used in the current study

Primer code / sequence (5'→3') [a]	Annealing temp (°C) [b]	Restriction enzyme (°C) [c]	Use of primers
**TAIL-PCR**	NA	NA	TAIL-PCR amplification
SP1: ATGCAACTTTCCAAAATTACTTTCGCTAT			
SP2: GGCAACACTGAGGTCGCAACCATCT			
SP3: GGCAGCGATGATTCCGACGCGTATT			
SP4: AATACGCGTCGGAATCATCGCTGCC			
SP5: AGATGGTTGCGACCTCAGTGTTGCC			
SP6: ATAGCGAAAGTAATTTTGGAAAGTTGCAT			
AD: STTGNTASTNCTNTGC			
**The key primer**			
***AvrPii*-J/C-CDS**	58	NA	J/C-CDS amplification
F: ATGCAACTTTCCAAAATTAC			
R: TTAGTTGCATTTATGATTA			
***AvrPii*-J-FL/GT**	58	NA/*Asc* I (37)	J-FL/GT amplification
F: TTGGCGCGCCTGGTAGATATCCGCTGAC			(*AvrPii*-J cloning)
R: TTGGCGCGCCCAGATTTGGAACTTTGGT			
***AvrPii*-J-RS**	58	NA	J-RS amplification
F: TGGTAGATATCCGCTGACTGG			(*AvrPii*-J resequencing)
R: TAGCCATTATCCAAGGGTTGCTCCACTCT			
***AvrPii*-J-MK1**	58	NA	J-MK1 amplification
F: GTATAATTCCTTTTCTTCCCTCCTT			(*AvrPii*-J genotyping)
R: CTAATTTAAATCGTGCGCTTTCAGA			
***AvrPii*-J-MK2**	58	NA	J-MK2 amplification
F: TGGTAGATATCCGCTGACTGG			(*AvrPii*-J genotyping)
R: CATATAATGCAATAGCGAAAGTAAT			
***AvrPii*-C-FL/RS**	58	NA	C-FL/RS amplification
F: AAGGCATAATAATTTCGTAAAAAGCGGTCTAA			(*AvrPii*-C resequencing)
R: ATGTATGCCTGGCCGTGACAATAACCC			
***AvrPii*-C-GT**	58	*Asc* I (37)	C-GT amplification
F: TTGGCGCGCCTCAGCGGATATTCACC			(*AvrPii*-C cloning)
R: TTGGCGCGCCTAGATTTTCAGGGTGC			
***AvrPii*-C-MK1**	58	NA	C-MK1 amplification
F: TCGTTATATTTCCATTGCTATTCAT			(*AvrPii*-C genotyping)
R: TAAAAATGAGTTAAATTATGCGTT			
***AvrPii*-C-MK2**	58	NA	C-MK2 amplification
F: CATAATAATTTCGTAAAAAGCGGTC			(*AvrPii*-C genotyping)
R: CATATAATGCAATAGCGAAAGTAAT			
**Domain swapping**			
**J_{pro}**	58	NA	J_{pro} amplification
F: TGGTAGATATCCGCTGAC			

R: AATTTTGGAGAGTTGCATTTTGGTAAATTGGAA			
**$C_{CDS+3'}$**<br>F: TTCCAATTTACCAAAATGCAACTCTCCAAAATT<br>R: TAGATTTTCAGGGTGC	58	NA	$C_{CDS+3'}$ amplification
**J_{pro}-F/$C_{CDS+3'}$-R**<br>F: TTGGCGCGCCTGGTAGATATCCGCTGAC<br>R: TTGGCGCGCCTAGATTTTCAGGGTGC	58	*Asc* I (37)	Mai1 construction
**C_{pro}**<br>F: TCAGCGGATATTCACC<br>R: AATTTTGGAAAGTTGCATTTTGGTAAGTTGGAA	58	NA	C-pro amplification
**$J_{CDS+3'}$**<br>F: TTCCAACTTACCAAAATGCAACTTTCCAAAATT<br>R: CAGATTTGGAACTTTGGT	58	NA	**$J_{CDS+3'}$** amplification
**C_{pro}-F/$J_{CDS+3'}$-R**<br>F: TTGGCGCGCCTCAGCGGATATTCACC<br>R: TTGGCGCGCCCAGATTTGGAACTTTGGT	58	*Asc* I (37)	Mai2 construction
**J/C-1**<br>F: CATTATATGCAGTCGGAATCGCAGCACTT<br>R: CAATAGCGAAAGTAATTTTGGAGAGTTGCAT	58	NA	Mai3 construction
**J/C-2**<br>F: CATCTCCGACGTTAAACTTGGACCCCGCA<br>R: GTTGCGACCTCAGTGTTGCCATTTAGGCAGGC	58	NA	Mai4 construction
**J/C-3**<br>F: ATGCGGCTTCGGCAGCGATGATTCC<br>R: TTGGAGCAATAATAATAAGTCTTGTCGC	58	NA	Mai5 construction
**$J_{pro+CDS}$**<br>F: TGGTAGATATCCGCTGAC<br>R: AGATATCAACTTACATTAGTTGCATTTATGA	58	NA	**$J_{pro+CDS}$** amplification
**$C_{3'}$**<br>F: TCATAAATGCAACTAATGTAAGTTGATATCT<br>R: TAGATTTTCAGGGTGC	58	NA	**$C_{3'}$** amplification
**$J_{pro+CDS}$-F/$C_{3'}$-R**<br>F: TTGGCGCGCCTGGTAGATATCCGCTGAC<br>R: TTGGCGCGCCTAGATTTTCAGGGTGC	58	*Asc* I (37)	Mai6 construction
**$C_{pro+CDS}$**<br>F: TCAGCGGATATTCACC<br>R: CAGATTTTAACTTACATTACTTGCACTTG	58	NA	**$C_{pro+CDS}$** amplification
**$J_{3'}$**<br>F: CAAGTGCAAGTAATGTAAGTTAAAATCTG<br>R: CAGATTTGGAACTTTGGT	58	NA	**$J_{3'}$** amplification
**$C_{pro+CDS}$-F/$J_{3'}$-R**<br>F: TTGGCGCGCCTCAGCGGATATTCACC	58	*Asc* I (37)	Mai7 construction

R: TTGGCGCGCCCAGATTTGGAACTTTGGT			
**Others**			
**HYG**<br>F: TTGGCTGGAGCTAGTGGAGGT<br>R: TCTGCTGCTCCATACAAGCCAAC	58	NA	Genotyping of transgenic progeny

The specific program for TAIL-PCR system

**Reaction round**	**Number of cycle**	**Cycle parameter**
I	1	94°C/2 min; 95°C/1 min.
	5	94°C/15 s; 62°C/1 min; 72°C/2 min.
	1	94°C/15 s; 25°C/3 min;<br>up to 72°C by 0. 2°C/s; 72°C/2 min.
	15	94°C/10 s; 62°C/1 min; 72°C/2 min;<br>94°C/10 s; 62°C/1 min; 72°C/2 min;<br>94°C/10 s; 44°C/1 min; 72°C 2 min.
	1	72°C/2 min.
II	15	94°C/10 s; 62°C/1 min; 72°C/2 min;<br>94°C/10 s; 62°C/1 min; 72°C/2 min;<br>94°C/10 s; 44°C/1 min; 72°C/2 min.
	1	72°C/2 min
III	15	94°C/10 s; 62°C/1 min; 72°C/2 min;<br>94°C/10 s; 62°C/1 min; 72°C/2 min;<br>94°C/10 s; 44°C/1 min; 72°C/2 min.
	1	72°C/5 min.

[a] For transformation test, sequence underlined was responding to the common restriction enzyme *Asc* I for ligating fragments (*AvrPii* and its mutants) into the binary vectors, pBHT2-AscI. F, forward; R, reverse.
[b] The regular PCR system was initiated by a 95°C/3 min denaturation, followed by 35 cycles of 95°C/15 s, 58°C/15 s, 72°C/1 min and a final extension step of 72°C/2 min. The Tail-PCR system was according to the specific program shown above. Amplicons were electrophoresed through a 1% agarose gel.
[c] The number shown in parentheses refers to the temperature at which the restriction digestion reaction was run.
