# Supplementary material for: The Lesson Learned from the Unique Evolutionary Story of Avirulence Gene AvrPii of Magnaporthe oryzae
Source: Genes (Basel). 2023 May 11;14(5):1065. doi: 10.3390/genes14051065 (PMC10218241; doi:10.3390/genes14051065)
Supplement: Supplementary file 1 [file genes-14-01065-s001.zip › genes-2373462-supplementary/23-5-7 Supplementary Materials for AvrPii/Table S2. Phenotypes, and genotypes of AvrPii.pdf]

Table S2. Genotypes and phenotypes of 377 isolates of *M. oryzae* characterized in the current study

						Genotype (1, 0) [d]					
Isolate	Cropping area [a]	Sampled rice cultivar / host	Sampled county	Sampled time [b]	Phenotype on *Pii* carrier [c]	*AvrPii*-C-MK1	*AvrPii*-C-MK2	*AvrPii*-J-MK1	*AvrPii*-J-MK2	*AvrPii* resequencing [e]	Integrated phenotype/genotype [f]
**Guangdong rice population (∑60)**						4	4	2	2	4C+2J	
CHL2345	GD I	Mabayinzhan	Qujiang	2008E	V	0	0	0	0		V/0
CHL2358	GD I	Shanyou 428	Wengyuan	2008E	V	0	0	0	0		V/0
CHL2370	GD II	Jinshanyou 3182	Longchuan	2008E	V	0	0	0	0		V/0
CHL2375	GD I	Shanyou 253	Renhua	2008E	V	0	0	0	0		V/0
CHL2384	GD I	Changguidao	Lianping	2008E	V	0	0	0	0		V/0
CHL2386	GD II	Fengsimiao	Gaoyao	2008E	V	0	0	0	0		V/0
CHL2387	GD II	Shanyou 428	Meixian	2008E	V	0	0	0	0		V/0
CHL2394	GD II	Nuo	Meixian	2008E	V	0	0	0	0		V/0
CHL2401	GD II	Huayougui 99	Wuhua	2008E	V	0	0	0	0		V/0
CHL2409	GD II	Zhongerruanzhan	Longmen	2008E	V	0	0	0	0		V/0
CHL2412	GD II	Shanyougui 99	Fengkai	2008E	A	**1**	1	0	0	✓	A/1C
CHL2416	GD II	Meixiangzhan	Xinxing	2008E	V	0	0	0	0		V/0
CHL2417	GD II	Tianyou 290	Xingning	2008E	V	0	0	0	0		V/0
CHL2419	GD II	Changguidao	Jiexi	2008E	V	0	0	0	0		V/0
CHL2422	GD III	Zaoyinzhan	Yangjiang	2008E	V	0	0	0	0		V/0
CHL2423	GD III	Sanyinian	Yangjiang	2008E	V	0	0	0	0		V/0
CHL2426	GD III	Youyou 389	Xinyi	2008E	V	0	0	0	0		V/0
CHL2433	GD III	Huayou 86	Xinyi	2008E	V	0	0	0	0		V/0
CHL2436	GD III	Huayou 86	Xinyi	2008E	V	0	0	0	0		V/0
CHL2438	GD III	Changguidao	Gaozhou	2008E	V	0	0	0	0		V/0
CHL2441	GD III	Zajiaodao	Gaozhou	2008E	V	0	0	0	0		V/0
CHL2442	GD III	Tianyou 998	Haifeng	2008E	V	0	0	0	0		V/0
CHL2444	GD III	Guangyinzhan	Foshan	2008E	V	0	0	0	0		V/0
CHL2446	GD III	Shuanghuangzhan	Jiedong	2008E	V	0	0	0	0		V/0
CHL2448	GD III	Teyou 721	Chaoan	2008E	V	0	0	0	0		V/0

CHL2449	GD II	Zengyouzhan	Zengcheng	2008E	V	0	0	0	0		V/0
CHL2452	GD III	Fengqin	Lianjiang	2008E	V	0	0	0			V/0
CHL2455	GD III	Bairizao	Huidong	2008E	V	0	0	0	0		V/0
CHL2457	GD III	Zhanyou 226	Huidong	2008E	V	0	0	0	0		V/0
CHL2458	GD III	Teyou 721	Yangchun	2008E	A	**1**	1	0	0	✓	A/1C
CHL2464	GD III	Taishanbai	Kaiping	2008E	V	0	0	0	0		V/0
CHL2467	GD I	Meixiangzhan	Renhua	2008L	V	0	0	0	0		V/0
CHL2488	GD I	Weixiang	Lechang	2008L	V	0	0	0	0		V/0
CHL2490	GD I	Guodao No.1	Ruyuan	2008L	V	0	0	0	0		V/0
CHL2491	GD I	Jinyou 207	Nanxiong	2008L	A	**1**	1	1	1	✓✓	A/1C_J
CHL2497	GD I	Meixiangzhan	Qujiang	2008L	A	**1**	1	0	0	✓	A/1C
CHL2507	GD I	Zayou	Wengyuan	2008L	V	0	0	0	0		V/0
CHL2513	GD I	Shanyou 46	Xinfeng	2008L	V	0	0	0	0		V/0
CHL2516	GD I	Shanyou 82	Jiaoling	2008L	V	0	0	0	0		V/0
CHL2518	GD II	Yuxiang 88	Longchuan	2008L	V	0	0	0	0		V/0
CHL2534	GD II	Shanyou 122	Meixian	2008L	V	0	0	0	0		V/0
CHL2548	GD II	Yixiang 3003	Dapu	2008L	V	0	0	0	0		V/0
CHL2549	GD II	Shanyou 122	Dapu	2008L	V	0	0	0	0		V/0
CHL2551	GD II	Boyouyun 3	Zijin	2008L	V	0	0	0	0		V/0
CHL2560	GD III	Huanghuazhan	Boluo	2008L	V	0	0	0	0		V/0
CHL2561	GD II	Longyou 665	Fougang	2008L	V	0	0	0	0		V/0
CHL2563	GD III	Zhanzinuo	Huidong	2008L	V	0	0	0	0		V/0
CHL2571	GD II	Qixinzhan	Longmen	2008L	V	0	0	0	0		V/0
CHL2581	GD II	Qiuyou 452	Qingxin	2008L	V	0	0	0	0		V/0
CHL2584	GD II	Zayounuo	Huaiji	2008L	V	0	0	0	0		V/0
CHL2586	GD II	Zhenguizhong	Yingde	2008L	V	0	0	0	0		V/0
CHL2593	GD II	Qiuyou 3008	Yunfu	2008L	V	0	0	0	0		V/0
CHL2600	GD II	Qiuyou 3008	Deqing	2008L	A	0	0	1	1	✓	A/1J
CHL2604	GD III	Guinongzhan	Haifeng	2008L	V	0	0	0	0		V/0
CHL2606	GD III	Xiaonongzhan	Zhuhai	2008L	V	0	0	0	0		V/0

CHL2608	GD III	Taiwanyouzhan	Yangchun	2008L	V	0	0	0	0		V/0
CHL2615	GD III	Bo II You 15	Lianjiang	2008L	V	0	0	0	0		V/0
CHL2626	GD III	Longyou 673	Xinyi	2008L	V	0	0	0	0		V/0
CHL2630	GD III	II You 084	Gaozhou	2008L	V	0	0	0	0		V/0
CHL2636	GD IV	Bo II You 15	Suixi	2008L	V	0	0	0	0		V/0
**Hunan population (∑60)**						23	23	21	21	23C+21J	
EHL0312	HN I	Tefu No.7	Taoyuan	2007	V	0	0	0	0		V/0
EHL0313	HN I	Tefu No.7	Taoyuan	2007	V	0	0	0	0		V/0
EHL0314	HN II	Weiyou 402	Anren	2007	A	**1**	1	0		✓	A/1C
EHL0317	HN II	Weiyou 402	Anren	2007	A	0	0	1	1	✓	A/1J
EHL0319	HN II	Liangyou 287	Liling	2007	A	**1**	1	1	1	✓✓	A/1C_J
EHL0321	HN II	V402	Hengnan	2007	A	**1**	1	0	0	✓	A/1C
EHL0323	HN II	V233	Hengdong	2007	A	**1**	1	0	0	✓	A/1C
EHL0324	HN II	I You 402	Hengdong	2007	A	**1**	1	0	0	✓	A/1C
EHL0326	HN II	I You 402	Hengnan	2007	A	**1**	1	0	0	✓	A/1C
EHL0327	HN II	Zhuliangyou 30	Dongan	2007	A	**1**	1	1	1	✓✓	A/1C_J
EHL0329	HN II	Douyuanyou 299	Dongan	2007	A	0	0	1	1	✓	A/1J
EHL0330	HN II	T You 99	Dongan	2007	A	0	0	1	1	✓	A/1J
EHL0331	HN II	Xianfeng 501	Dongan	2007	V	0	0	0	0		V/0
EHL0337	HN II	Jinyou 71	Dongan	2007	V	0	0	0	0		V/0
EHL0338	HN II	Jinyou 898	Dongan	2007	A	**1**	1	1	1	✓✓	A/1C_J
EHL0339	HN II	Nongping 001	Dongan	2007	V	0	0	0	0		V/0
EHL0340	HN II	Weiyou 507	Dongan	2007	A	0	0	1	1	✓	A/1J
EHL0341	HN II	Weiyou 298	Dongan	2007	A	**1**	1	0	0	✓	A/1C
EHL0342	HN II	Weiyou 463	Dongan	2007	A	0	0	1	1	✓	A/1J
EHL0345	HN II	T You 213	Dongan	2007	V	0	0	0	0		V/0
EHL0346	HN II	T You 103	Dongan	2007	A	**1**	1	0	0	✓	A/1C
EHL0347	HN II	T You 404	Dongan	2007	V	0	0	0	0		V/0
EHL0349	HN II	Jinyou 402	Dongan	2007	V	0	0	0	0		V/0
EHL0350	HN II	Xianfeng No.1	Dongan	2007	V	0	0	0	0		V/0
EHL0353	HN II	Nongfengyou 909	Dongan	2007	A	0	0	1	1	✓	A/1J

EHL0354	HN II	Jinyou 821	Dongan	2007	A	0	0	1	1	✓	A/1J
EHL0355	HN II	Weiyou 463	Dongan	2007	A	0	0	1	1	✓	A/1J
EHL0356	HN II	Zhongyou No.1	Dongan	2007	A	**1**	1	1	1	✓✓	A/1C_J
EHL0360	HN II	Zhongyou No.1	Dongan	2007	A	**1**	1	1	1	✓✓	A/1C_J
EHL0361	HN II	Zhuliangyou 02	Dongan	2007	A	**1**	1	1	1	✓✓	A/1C_J
EHL0363	HN II	Xiangnuo	Dongan	2007	A	**1**	1	0	0	✓	A/1C
EHL0364	HN II	T You 706	Dongan	2007	A	0	0	1	1	✓	A/1J
EHL0365	HN II	Zhongyou 493	Dongan	2007	V	0	0	0	0		V/0
EHL0367	HN II	Jinyou 463	Dongan	2007	V	0	0	0	0		V/0
EHL0368	HN II	Zhuliangyou 83	Dongan	2007	V	0	0	0	0		V/0
EHL0369	HN III	Jinyou 899	Daoxian	2007	V	0	0	0	0		V/0
EHL0370	HN III	Jinyou 463	Daoxian	2007	A	**1**	1	1	1	✓✓	A/1C_J
EHL0372	HN III	Nuogu	Daoxian	2007	V	0	0	0	0		V/0
EHL0373	HN III	Jinyou 463	Daoxian	2007	A	**1**	1	1	1	✓✓	A/1C_J
EHL0374	HN III	Shennongdao 101	Daoxian	2007	A	**1**	1	1	1	✓✓	A/1C_J
EHL0377	HN I	Jing No.5	Yiyang	2007	A	**1**	1	0	0	✓	A/1-C
EHL0378	HN I	Wanxiang No. 13	Taojiang	2007	A	**1**	1	1	1	✓✓	A/1C_J
EHL0379	HN I	Pin No. 3	Taojiang	2007	V	0	0	0	0		V/0
EHL0380	HN I	Wannuo	Yiyang	2007	V	0	0	0	0		V/0
EHL0381	HN I	Wannuo	Yiyang	2007	V	0	0	0	0		V/0
EHL0382	HN I	Nuogu	YueYang	2007	A	**1**	1	1	1	✓✓	A/1C_J
EHL0385	HN II	II You 58	Liuyang	2007	V	0	0	0	0		V/0
EHL0386	HN II	Minghui 63	Liuyang	2007	V	0	0	0	0		V/0
EHL0387	HN II	II You 838	Liuyang	2007	V	0	0	0	0		V/0
EHL0393	HN II	Y NO. 232	Youxian	2007	A	**1**	1	0	0	✓	A/1C
EHL0394	HN II	Xinshanyou 64	Anren	2007	A	**1**	1	1	1	✓✓	A/1C_J
EHL0395	HN IV	Zhongyou 288	Huaihua	2007	V	0	0	0	0		V/0
EHL0963	HN I	Xianghuayou No. 7	Taojiang	2008	A	**1**	1	0	0	✓	A/1C
EHL0975	HN I	Fuyou II	Taojiang	2008	V	0	0	0	0		V/0
EHL0979	HN II	Mixiang No.1	Changsha	2008	V	0	0	0	0		V/0
EHL0982	HN II	Xiangwanxian No.17	Changsha	2008	V	0	0	0	0		V/0
EHL0987	HN II	Chaojidao 527	Zhuzhou	2008	V	0	0	0	0		V/0

EHL0988	HN II	Chaojidao 527	Zhuzhou	2008	V	0	0	0	0		V/0
EHL0989	HN II	Xiangwanxian No.13	Zhuzhou	2008	V	0	0	0	0		V/0
EHL0991	HN II	Xiangzaoxian 24	Liling	2008	V	0	0	0	0		V/0
**Guizhou population (∑60)**						17	17	5	5	17C+5J	
CHL583	GZ III	Chujing	Panxian	2001	A	**1**	1	0	0	✓	A/1C
CHL584	GZ I	Bendinuo	Danzhai	2001	A	**1**	1	0	0	✓	A/1C
CHL586	GZ VI	Baikejing	Nayong	2001	A	**1**	1	0	0	✓	A/1C
CHL588	GZ I	Aizinuo	Huaxi	2001	V	0	0	0	0		V/0
CHL589	GZ I	K You 5	Dushan	2001	V	0	0	0	0		V/0
CHL590	GZ I	Bendigaonuo	Huaxi	2001	A	**1**	1	0	0	✓	A/1C
CHL591	GZ I	Nuodao	Dushan	2001	A	**1**	1	0	0	✓	A/1C
CHL592	GZ I	Nuodao	Xifeng	2001	V	0	0	0	0		V/0
CHL593	GZ III	Nuodao	Panxian	2001	V	0	0	0	0		V/0
CHL594	GZ I	Nuodao	Xifeng	2001	V	0	0	0	0		V/0
CHL597	GZ VI	Baikejing	Nayong	2001	A	**1**	1	0	0	✓	A/1C
CHL598	GZ VI	Bijing 37	Nayong	2001	V	0	0	0	0		V/0
CHL599	GZ III	Nuodao	Xingren	2001	A	**1**	1	0	0	✓	A/1C
CHL601	GZ III	Gangyou 527	Xingyi	2001	V	0	0	0	0		V/0
CHL602	GZ I	Xieyou No.6	Dushan	2001	V	0	0	0	0		V/0
CHL605	GZ III	Gangyou 725	Xingyi	2001	V	0	0	0	0		V/0
CHL610	GZ I	D You 68	Kaiyang	2001	V	0	0	0	0		V/0
CHL611	GZ I	Jinwan 3	Huaxi	2001	A	0	0	1	1	✓	A/1J
CHL612	GZ VI	Maomaonuo	Nayong	2001	A	**1**	1	0	0	✓	A/1C
CHL614	GZ VI	800 Li	Nayong	2001	V	0	0	0	0		V/0
CHL615	GZ VI	84-15	Nayong	2001	A	**1**	1	0	0	✓	A/1C
CHL616	GZ VI	800 Li	Nayong	2001	V	0	0	0	0		V/0
CHL617	GZ VI	84-15	Nayong	2001	A	**1**	1	0	0	✓	A/1C
CHL618	GZ III	Gangyou 725	Xingren	2001	V	0	0	0	0		V/0
CHL621	GZ I	Feiduo No.1	Zunyi	2001	V	0	0	0	0		V/0
CHL622	GZ I	Nuodao	Xiuwen	2001	V	0	0	0	0		V/0
CHL624	GZ I	Xinxiangyou 80	Xiuwen	2001	A	0	0	1	1	✓	A/1J
CHL625	GZ II	Unkown	Tianzhu	2001	A	0	0	1	1	✓	A/1J

CHL626	GZ I	Nuodao	Kaiyang	2001	A	**1**	1	0	0	✓	A/1C
CHL627	GZ I	Bendinuo	Qingzhen	2001	A	**1**	1	0	0	✓	A/1C
CHL629	GZ IV	Liangyou 6078	Liping	2001	V	0	0	0	0		V/0
CHL630	GZ III	Manbaxiang	Xingyi	2001	V	0	0	0	0		V/0
CHL639	GZ III	Gangyou 725	Xingren	2001	V	0	0	0	0		V/0
CHL640	GZ III	Guihuanuo	Xingyi	2001	V	0	0	0	0		V/0
CHL641	GZ I	Xinxiangyou 80	Xiuwen	2001	A	0	0	1	1	✓	A/1J
CHL643	GZ I	Unkown	Yuqing	2001	V	0	0	0	0		V/0
CHL644	GZ II	79-16	Sinan	2001	A	**1**	1	0	0	✓	A/1C
CHL645	GZ II	Baiyangnuo	Sinan	2001	A	**1**	1	0	0	✓	A/1C
CHL646	GZ II	Gangyou 22	Sinan	2001	V	0	0	0	0		V/0
CHL648	GZ II	Gangyou 22	Sinan	2001	V	0	0	0	0		V/0
CHL649	GZ II	Baiyangnuo	Sinan	2001	A	**1**	1	0	0	✓	A/1C
CHL650	GZ I	K77	Zunyi	2001	V	0	0	0	0		V/0
CHL652	GZ I	K77	Zunyi	2001	A	**1**	1	1	1	✓✓	A/1C_J
CHL653	GZ II	79-16	Sinan	2001	V	0	0	0	0		V/0
CHL654	GZ I	Feiduo No.1	Zunyi	2001	V	0	0	0	0		V/0
CHL655	GZ VI	Maomaonuo	Nayong	2001	A	**1**	1	0	0	✓	A/1C
CHL656	GZ I	Liangyou 63	Taijiang	2001	V	0	0	0	0		V/0
CHL657	GZ II	Shanyou 73	Jianhe	2001	V	0	0	0	0		V/0
CHL658	GZ I	Teyou 63	Dushan	2001	V	0	0	0	0		V/0
CHL659	GZ I	Xieyou 63	Dushan	2001	V	0	0	0	0		V/0
CHL661	GZ I	Shanyou 63	Huangping	2001	V	0	0	0	0		V/0
CHL662	GZ III	D You 527	Xingyi	2001	V	0	0	0	0		V/0
CHL665	GZ III	Gangyou	Xingyi	2001	V	0	0	0	0		V/0
CHL666	GZ I	Shanyou 63	Kaiyang	2001	V	0	0	0	0		V/0
CHL667	GZ III	Teyou 559	Xingyi	2001	V	0	0	0	0		V/0
CHL668	GZ I	Jinyou 63	Kaiyang	2001	V	0	0	0	0		V/0
CHL670	GZ I	Gangyou 22	Dushan	2001	V	0	0	0	0		V/0
CHL672	GZ IV	Shanyou 63	Congjiang	2001	V	0	0	0	0		V/0
CHL674	GZ III	Guihuanuo	Xingyi	2001	V	0	0	0	0		V/0
CHL675	GZ III	Kekexiang	Xingyi	2001	V	0	0	0	0		V/0

**Liaoning population (∑60)**						24	24	0	0	24C+0J	
EHL0396	LN I	Minxi 12	Zhuanghe	2006	V	0	0	0	0		V/0
EHL0397	LN I	Chenhong 59	Zhuanghe	2006	A	**1**	1	0	0	✓	A/1C
EHL0400	LN I	Lige	Zhuanghe	2006	V	0	0	0	0		V/0
EHL0409	LN I	Yanjing 167	Donggang	2006	V	0	0	0	0		V/0
EHL0412	LN I	Huanghai No.6	Donggang	2006	V	0	0	0	0		V/0
EHL0431	LN I	Liaodan 203	Donggang	2006	V	0	0	0	0		V/0
EHL0433	LN I	Liaohan 109	Donggang	2006	A	**1**	1	0	0	✓	A/1C
EHL0444	LN II	Chenhong 59	Pulandian	2006	V	0	0	0	0		V/0
EHL0445	LN II	Minxi 12	Pulandian	2006	A	**1**	1	0	0	✓	A/1C
EHL0446	LN II	Gangyu 129	Pulandian	2006	A	**1**	1	0	0	✓	A/1C
EHL0447	LN II	Koshihikari	Pulandian	2006	A	**1**	1	0	0	✓	A/1C
EHL0468	LN II	Maoyang No.3	Fengchen	2006	V	0	0	0	0		V/0
EHL0470	LN II	Maoyang 3	Fengchen	2006	V	0	0	0	0		V/0
EHL0472	LN II	Panjin 78-2	Fengchen	2006	A	**1**	1	0	0	✓	A/1C
EHL0474	LN II	Chenhe No.1	Fengchen	2006	A	**1**	1	0	0	✓	A/1C
EHL0484	LN II	Yuanfeng No.9	Fushun	2006	V	0	0	0	0		V/0
EHL0486	LN II	Fuyou 504	Fushun	2006	V	0	0	0	0		V/0
EHL0489	LN II	Shennong 0705	Fushun	2006	A	**1**	1	0	0	✓	A/1C
EHL0491	LN II	Jijing 88	Fushun	2006	A	**1**	1	0	0	✓	A/1C
EHL0492	LN II	Xin No.2	Fushun	2006	V	0	0	0	0		V/0
EHL0493	LN II	Shennong 315	Fushun	2006	V	0	0	0	0		V/0
EHL0496	LN II	Su 01-18	Dashiqiao	2006	A	**1**	1	0	0	✓	A/1C
EHL0498	LN II	Yanfeng	Dashiqiao	2006	A	**1**	1	0	0	✓	A/1C
EHL0499	LN II	Yanfeng 47-12	Dashiqiao	2006	A	**1**	1	0	0	✓	A/1C
EHL0500	LN III	Chenhe No.1	Panjin	2006	A	**1**	1	0	0	✓	A/1C
EHL0503	LN III	Maoyang No.3	Panjin	2006	A	**1**	1	0	0	✓	A/1C
EHL0505	LN III	Liaohe 12	Panjin	2006	A	**1**	1	0	0	✓	A/1C
EHL0509	LN III	Yanjing 228	Panjin	2006	V	0	0	0	0		V/0
EHL0515	LN III	Liaohe No.1	Panjin	2006	A	**1**	1	0	0	✓	A/1C
EHL0517	LN III	Huadan 995	Shenyang	2006	A	**1**	1	0	0	✓	A/1C
EHL0519	LN III	Huadan 995	Tieling	2006	A	**1**	1	0	0	✓	A/1C

EHL0879	LN I	Liaoxing No.1	Dandong	2007	V	0	0	0	0		V/0
EHL0880	LN I	Gangyu No.2	Dandong	2007	V	0	0	0	0		V/0
EHL0882	LN I	Chenhe No.1	Dandong	2007	V	0	0	0	0		V/0
EHL0883	LN I	Maoyang No.5	Dandong	2007	A	**1**	1	0	0	✓	A/1C
EHL0885	LN I	Liaojing No.9	Dandong	2007	A	**1**	1	0	0	✓	A/1C
EHL0916	LN II	Heixiangdao	Dalian	2007	V	0	0	0	0		V/0
EHL0917	LN II	Zhuangyan No.8	Dalian	2007	V	0	0	0	0		V/0
EHL0918	LN II	Koshihikari	Dalian	2007	A	**1**	1	0	0	✓	A/1C
EHL0919	LN II	Yanfeng 47	Dalian	2007	V	0	0	0	0		V/0
EHL0921	LN II	Gangyuan No.8	Dalian	2007	V	0	0	0	0		V/0
EHL0922	LN II	Liaonong 06-7	Qinyuan	2007	V	0	0	0	0		V/0
EHL0925	LN II	Fuxing 90	Qinyuan	2007	V	0	0	0	0		V/0
EHL0926	LN II	Jijing 88	Qinyuan	2007	V	0	0	0	0		V/0
EHL0928	LN II	Liaonong 06-7	Xinbin	2007	V	0	0	0	0		V/0
EHL0929	LN II	Fu 9906	Xinbin	2007	V	0	0	0	0		V/0
EHL0930	LN II	Xinyi 3	Xinbin	2007	V	0	0	0	0		V/0
EHL0932	LN II	Xinyu No.3	Xinbin	2007	V	0	0	0	0		V/0
EHL0934	LN II	Xinyu 4	Xinbin	2008	V	0	0	0	0		V/0
EHL0935	LN II	Jijing 88	Xinbin	2007	A	**1**	1	0	0	✓	A/1C
EHL0936	LN II	Shendao 37	Xifeng	2007	V	0	0	0	0		V/0
EHL0937	LN II	Shennong 315	Xifeng	2007	V	0	0	0	0		V/0
EHL0939	LN II	Xinyi No.3	Xifeng	2007	V	0	0	0	0		V/0
EHL0940	LN II	Fuxing 90	Xifeng	2007	V	0	0	0	0		V/0
EHL0941	LN II	Xinyi No.3	Kaiyuan	2007	V	0	0	0	0		V/0
EHL0942	LN II	Shennong 9903	Kaiyuan	2007	A	**1**	1	0	0	✓	A/1C
EHL0943	LN II	Huadan No.995	Kaiyuan	2007	V	0	0	0	0		V/0
EHL0944	LN II	Fuxing 90	Kaiyuan	2007	V	0	0	0	0		V/0
EHL0961	LN IV	Chenhe No.1	Suizhong	2007	V	0	0	0	0		V/0
EHL0962	LN IV	Yanjing 218	Suizhong	2007	A	**1**	1	0	0	✓	A/1C
**Jilin population (∑60)**						8	8	0	0	8C+0J	
CHL678	JL I	Tong 31	Yongji	2003	V	0	0	0	0		V/0
CHL680	JL I	Ji 04	Yongji	2003	V	0	0	0	0		V/0

CHL682	JL I	Tong 313	Yongji	2003	V	0	0	0	0		V/0
CHL684	JL I	Fengyou 201	Yongji	2003	V	0	0	0	0		V/0
CHL686	JL I	Pingjing 1	Yongji	2003	V	0	0	0	0		V/0
CHL688	JL I	Jiu 01 C 6	Yongji	2003	V	0	0	0	0		V/0
CHL690	JL I	Changbai 9	Shulan	2003	A	**1**	1	0	0	✓	A/1C
CHL692	JL I	Dongnong V 7	Shulan	2003	V	0	0	0	0		V/0
CHL693	JL I	Yan 312	Shulan	2003	V	0	0	0	0		V/0
CHL695	JL I	Songjing 6	Shulan	2003	V	0	0	0	0		V/0
CHL697	JL I	301	Shulan	2003	V	0	0	0	0		V/0
CHL699	JL I	Tongyu 12	Shulan	2003	V	0	0	0	0		V/0
CHL700	JL I	Wuyou 1	Shulan	2003	V	0	0	0	0		V/0
CHL701	JL I	99-35	Panshi	2003	V	0	0	0	0		V/0
CHL702	JL I	Tongfeng No. 3	Panshi	2003	V	0	0	0	0		V/0
CHL703	JL I	Ji 01-3628	Panshi	2003	V	0	0	0	0		V/0
CHL704	JL I	Pingjing No. 2	Panshi	2003	V	0	0	0	0		V/0
CHL705	JL I	Chang 6	Panshi	2003	V	0	0	0	0		V/0
CHL708	JL IV	Tongjing 612	Jiaohe	2003	V	0	0	0	0		V/0
CHL710	JL IV	Yan 313	Jiaohe	2003	V	0	0	0	0		V/0
CHL712	JL IV	Changbai 9	Jiaohe	2003	V	0	0	0	0		V/0
CHL713	JL IV	Jinlang No. 1	Jiaohe	2003	V	0	0	0	0		V/0
CHL714	JL IV	Wuyou C	Jiaohe	2003	V	0	0	0	0		V/0
CHL719	JL IV	124	Jiaohe	2003	A	1	1	0	0	✓	A/1C
CHL718	JL IV	Changlixiang 12	Jiaohe	2003	V	0	0	0	0		V/0
CHL721	JL I	Jiu 01 D 13	Jilin	2003	V	0	0	0	0		V/0
CHL722	JL I	Ji T620-4	Jilin	2003	V	0	0	0	0		V/0
CHL724	JL I	T 02	Jilin	2003	V	0	0	0	0		V/0
CHL725	JL I	Tongjing 790 B	Jilin	2003	V	0	0	0	0		V/0
CHL730	JL I	Tongnian 3	Jilin	2003	V	0	0	0	0		V/0
CHL731	JL I	Tongyu 308	Jilin	2003	V	0	0	0	0		V/0
CHL733	JL I	Tongnian 23	Meihekou	2003	V	0	0	0	0		V/0
CHL734	JL I	Tong 3158	Liuhe	2003	V	0	0	0	0		V/0
CHL735	JL I	Daliwang	Liuhe	2003	V	0	0	0	0		V/0

CHL736	JL I	Unknown	Lishu	2003	V	0	0	0	0		V/0
CHL737	JL I	Unknown	Yitong	2003	V	0	0	0	0		V/0
CHL739	JL I	182	Jiutai	2003	V	0	0	0	0		V/0
CHL740	JL I	183	Jiutai	2003	V	0	0	0	0		V/0
CHL741	JL I	No. 55	Jiutai	2003	A	**1**	1	0	0	✓	A/1C
CHL742	JL I	No. 55	Jiutai	2003	A	**1**	1	0	0	✓	A/1C
CHL743	JL I	No. 52	Jiutai	2003	V	0	0	0	0		V/0
CHL744	JL I	Tong 793	Dehui	2003	V	0	0	0	0		V/0
CHL745	JL I	Tong 190	Dehui	2003	V	0	0	0	0		V/0
CHL748	JL I	Changxiangsi	Dongfeng	2003	V	0	0	0	0		V/0
CHL749	JL I	Tongjing 769	Dongfeng	2003	A	**1**	1	0	0	✓	A/1C
CHL750	JL I	307	Dongfeng	2003	V	0	0	0	0		V/0
CHL751	JL I	Tongyin 58	Dongfeng	2003	V	0	0	0	0		V/0
CHL752	JL I	Zupei 11	Dongfeng	2003	V	0	0	0	0		V/0
CHL754	JL I	Chaochan 2	Gongzhuling	2003	V	0	0	0	0		V/0
CHL755	JL I	Unknown	Yitong	2003	V	0	0	0	0		V/0
CHL756	JL I	Xueguang	Yongji	2002	V	0	0	0	0		V/0
CHL757	JL IV	Unknown	Jiaohe	2002	V	0	0	0	0		V/0
CHL759	JL III	Shangyu No. 397	Helong	2002	V	0	0	0	0		V/0
CHL760	JL I	Menggudao	Meihekou	2002	V	0	0	0	0		V/0
CHL761	JL I	403	Liuhe	2002	V	0	0	0	0		V/0
CHL762	JL I	112	Liuhe	2002	V	0	0	0	0		V/0
CHL764	JL I	Tong 458	Dongfeng	2002	A	**1**	1	0	0	✓	A/1C
CHL765	JL I	Unknown	Gongzhuling	2002	V	0	0	0	0		V/0
CHL766	JL I	Unknown	Gongzhuling	2002	A	**1**	1	0	0	✓	A/1C
CHL767	JL I	939	Baichen	2002	A	**1**	1	0	0	✓	A/1C
**Heilongjiang population (∑60)**						6	6	0	0	6C+0J	
EHL0524	HLJ III	Songjing 7	Zhaoyuan	2006	A	**1**	1	0	0	✓	A/1C
EHL0527	HLJ II	Kendao 10	Anqin	2006	A	**1**	1	0	0	✓	A/1C
EHL0532	HLJ II	Song 98-131	Anqin	2006	V	0	0	0	0		V/0
EHL0540	HLJ I	Songjing 10	Achen	2006	V	0	0	0	0		V/0
EHL0547	HLJ II	Kendao 12	Tonghe	2006	V	0	0	0	0		V/0

EHL0555	HLJ II	Kuyuku 131	Tonghe	2006	V	0	0	0	0		V/0
EHL0557	HLJ II	Suijing No.4	Tonghe	2006	V	0	0	0	0		V/0
EHL0559	HLJ II	Dongnong 220	Shangzhi	2006	V	0	0	0	0		V/0
EHL0560	HLJ II	Puyou 18	Shangzhi	2006	V	0	0	0	0		V/0
EHL0567	HLJ II	Wuyoudao No.3	Shangzhi	2006	V	0	0	0	0		V/0
EHL0568	HLJ II	Wuyoudao No.3	Shangzhi	2006	V	0	0	0	0		V/0
EHL0570	HLJ II	Mudanjiang 19	Shangzhi	2006	V	0	0	0	0		V/0
EHL0571	HLJ II	Kendao 10	Shangzhi	2006	V	0	0	0	0		V/0
EHL0592	HLJ II	Shangyu 397	Muling	2006	V	0	0	0	0		V/0
EHL0598	HLJ III	Punian No.7	Hulin	2006	V	0	0	0	0		V/0
EHL0599	HLJ III	Kenjiandao No.6	Hulin	2006	V	0	0	0	0		V/0
EHL0605	HLJ III	Kuyuku 131	Hulin	2006	V	0	0	0	0		V/0
EHL0611	HLJ III	Kendao 12	Hulin	2006	V	0	0	0	0		V/0
EHL0612	HLJ II	Kuyuku 131	Mishan	2006	V	0	0	0	0		V/0
EHL0615	HLJ II	Suijing No.4	Mishan	2006	V	0	0	0	0		V/0
EHL0619	HLJ II	Kuyuku 131	Mishan	2006	V	0	0	0	0		V/0
EHL0622	HLJ II	Longdun 02-318	Jidong	2006	V	0	0	0	0		V/0
EHL0624	HLJ II	Kuyuku 131	Tangyuan	2006	A	**1**	1	0	0	✓	A/1C
EHL0625	HLJ II	Kuyuku 131	Huachuan	2006	V	0	0	0	0		V/0
EHL0629	HLJ II	Longdun 102	Jiamusi	2006	V	0	0	0	0		V/0
EHL0633	HLJ II	Kuyuku 131	Jiamusi	2006	V	0	0	0	0		V/0
EHL0634	HLJ II	Kuyuku 131	Baoqin	2006	V	0	0	0	0		V/0
EHL0637	HLJ II	Kuyuku 131	Jixian	2006	A	**1**	1	0	0	✓	A/1C
EHL0750	HLJ I	Kuyuku 131	Mudanjiang	2006	V	0	0	0	0		V/0
EHL0755	HLJ II	Kuyuku 131	Tangyuan	2006	A	**1**	1	0	0	✓	A/1C
EHL0757	HLJ II	Kendao No.6	Tangyuan	2006	V	0	0	0	0		V/0
EHL0764	HLJ II	Kendao No.11	Qinan	2006	V	0	0	0	0		V/0
EHL0766	HLJ II	Kuyuku 131	Qinan	2006	V	0	0	0	0		V/0
EHL0770	HLJ II	Longjing 17	Qinan	2006	V	0	0	0	0		V/0
EHL0776	HLJ I	Kendao No.6	Mudanjiang	2006	V	0	0	0	0		V/0
EHL0779	HLJ I	Kuyuku 131	Mudanjiang	2006	V	0	0	0	0		V/0
EHL0781	HLJ I	Longjing 17	Mudanjiang	2006	V	0	0	0	0		V/0

EHL0787	HLJ III	Kenjiandao 13	Jiansanjiang	2006	V	0	0	0	0		V/0
EHL0790	HLJ III	Kuyuku 131	Jiansanjiang	2006	V	0	0	0	0		V/0
EHL0794	HLJ I	Jite 639	Wuchang	2007	A	**1**	1	0	0	✓	A/1C
EHL0798	HLJ III	Songjing 3	Zhaodong	2007	V	0	0	0	0		V/0
EHL0801	HLJ III	Songjing 12	Zhaodong	2007	V	0	0	0	0		V/0
EHL0814	HLJ III	Jijing 106	Zhaodong	2007	V	0	0	0	0		V/0
EHL0821	HLJ I	Songjing 5	Shuangchen	2007	V	0	0	0	0		V/0
EHL0828	HLJ I	Songjing 6	Shuangchen	2007	V	0	0	0	0		V/0
EHL0830	HLJ II	Kendao 12	Fangzheng	2007	V	0	0	0	0		V/0
EHL0834	HLJ I	Menggudao	Mudanjiang	2007	V	0	0	0	0		V/0
EHL0836	HLJ I	Shangyu 397	Mudanjiang	2007	V	0	0	0	0		V/0
EHL0837	HLJ I	Shangyu 397	Mudanjiang	2007	V	0	0	0	0		V/0
EHL0838	HLJ I	Shangyu 397	Mudanjiang	2007	V	0	0	0	0		V/0
EHL0849	HLJ II	Kuyuku 131	Muling	2007	V	0	0	0	0		V/0
EHL0850	HLJ II	Kuyuku 131	Muling	2007	V	0	0	0	0		V/0
EHL0856	HLJ II	Puyou 9	Muling	2007	V	0	0	0	0		V/0
EHL0858	HLJ II	Puyou 9	Muling	2007	V	0	0	0	0		V/0
EHL0864	HLJ II	Unkown	Jidong	2007	V	0	0	0	0		V/0
EHL0866	HLJ III	Longjing 17	Hulin	2007	V	0	0	0	0		V/0
EHL0868	HLJ III	Kenjiandao 10	Hulin	2007	V	0	0	0	0		V/0
EHL0869	HLJ III	Kenjiandao 10	Hulin	2007	V	0	0	0	0		V/0
EHL0870	HLJ III	Longjing 17	Hulin	2007	V	0	0	0	0		V/0
EHL0871	HLJ III	Longjing 17	Hulin	2007	V	0	0	0	0		V/0
**Outgroup (weed-derived isolates) (∑17)**						0	0	7		0C+7J	
CHL325	N/A	Weed	Guangzhou	2001	ALL A	0	0	0	0		A/0
CHL326	N/A	Weed	Guangzhou	2001	ALL A	0	0	0	0		A/0
CHL329	N/A	Weed	Guangzhou	2001	ALL A	0	0	0	0		A/0
CHL330	N/A	Weed	Guangzhou	2001	ALL A	0	0	0	0		A/0
CHL331	N/A	Weed	Guangzhou	2001	ALL A	0	0	0	0		A/0
CHL332	N/A	Weed	Guangzhou	2001	ALL A	0	0	0	0		A/0
EHL2032	N/A	Weed	Hainan	2016	ALL A	0	0	1	1	✓	A/1J
EHL2034	N/A	Weed	Hainan	2016	ALL A	0	0	1	1	✓	A/1J

EHL2040	N/A	Weed	Hainan	2016	ALL A	0	0	1	1	✓	A/1J
EHL2042	N/A	Weed	Hainan	2016	ALL A	0	0	1	1	✓	A/1J
EHL2046	N/A	Weed	Hainan	2016	ALL A	0	0	0	0		A/0
EHL2051	N/A	Weed	Hainan	2017	ALL A	0	0	1	1	✓	A/1J
EHL2052	N/A	Weed	Hainan	2017	ALL A	0	0	1	1	✓	A/1J
EHL2053	N/A	Weed	Hainan	2017	ALL A	0	0	0	0		A/0
EHL2054	N/A	Weed	Hainan	2017	ALL A	0	0	0	0		A/0
EHL2055	N/A	Weed	Hainan	2017	ALL A	0	0	0	0		A/0
EHL2056	N/A	Weed	Hainan	2017	ALL A	0	0	1	1	✓	A/1J

[a] The rice-growing areas in the respective provinces, China, were adopted from Regionalization of Rice Cropping in China Edited in 1988 by China National Rice Research Institute, Hangzhou, China (45); and GD I and GD II mean #1 and #2 rice-growing areas, respectively, in Guangdong province and so on; N/A, data not available.

[b] 2008E and 2008L mean Early season (from March to July) and Late season (from July to November) in 2008, respectively and so on.

[c] Phenotype was derived from the *Pii* carrier, IRBLi-F5; A, avirulent; V, virulent; All A, those could not infect the *Pii* carrier, as well as several susceptible check cultivars thereby calling them as non-rice pathogen.

[d] PCR product-based genotypes 0, 1, respectively, indicate absence and presence of amplicon detected.

[e] Isolates marked by ✓ and ✓ ✓, respectively, indicate one and two copy/copies, those were subjected to resequencing.

[f] The integrated phenotype/genotypes, A/0 and V/0, respectively, indicate non-rice pathogen, and rice pathogen lack of *AvrPii*; A/1C, A/1J, and A1C_J, respectively, the rice pathogen isolates carried C, J, and C_J copy/copies those were avirulent on the *Pii* carrier.
