# Supplementary material for: The Lesson Learned from the Unique Evolutionary Story of Avirulence Gene AvrPii of Magnaporthe oryzae
Source: Genes (Basel). 2023 May 11;14(5):1065. doi: 10.3390/genes14051065 (PMC10218241; doi:10.3390/genes14051065)
Supplement: Supplementary file 1 [file genes-14-01065-s001.zip › genes-2373462-supplementary/23-5-7 Supplementary Materials for AvrPii/Table S3. The FL sequemces of AvrPii mutants.pdf]

Table S3. The full-length sequences of seven haplotype-chimeric mutants of the *AvrPii* family (sequence of *AvrPii*-J was in bule, and that of *AvrPii*-C in red; the coding sequences were in backgroud color; also see **Fig. 2**)

Mai1 (*AvrPii*-J_{pro}/$C_{CDS+3'}$)

TGGTAGATATCCGCTGACTGGGGGCAACATTGGTTGGATTTTAAAGTACATATATAGAAACGAAAATAAGTGGAAAAA
GAAGAACAAGAGTAAGGGTTATTAATTACACGACTAAATGTGGTAATTTAGCTATGTTATAACCAGGCAAGCACTACA
AATAGCCCATGGCAATCAGGAAGAGGCCGATATGTTACGATTGAATAATAAAATATTTTATAAACTTGCATTTGGATG
TAAATTGTAAATTATGCATTTGAGCTCCTCTGTTTCTATTAACAGAAATGTTTTTAAACAAGGTACCTAAATGCAATT
CTATATTTTTTATGCGGATTTATGCAGGCCCAAATCCGTAGGAAATACTATTAGTTATAATAATATAAAAACCTCGGCT
TCTTGTATATTACTGTATAATTCCTTTTCTTCCCTCCTTATATTTTTCATCTTAAAACTAAACATTTAAATTCAGCTTC
AAATATACCAAAATCCCCTTTTATTCCTTCCAATTTACCAAAATGCAACTCTCCAAAATTACTTTCGCTATTGCATTAT
ATGCAGTCGGAATCGCAGCACTTCCCACTCCGGCCTGCCTAAATGGCAACACTGAGGTCGCAACCATCTCCGACGTTA
AACTTGGACCCCGCAGCGACAAGACTTATTATTATTGCTCCAAATGCGGCTTCGGCAGCAATGATTCCGACACGTATT
TTAATCACAAGTGCAAGTAATGTAAGTTGATATCTAAACGCATAATTTAACTCATTTTTAAAAAAATAAATTATTCTA
ACGTTTAATTATAATGCATACGAATTAAACAGCATTTTTCAAGCGCGTGACGGACAGTTATGGATTCTGCAAATTTTA
AATACCCGGTGTCACGGCCAGGCATATATCGGAATGTGAGGATCAGGTCCCCACACCTACCCTCAGAATTACGACTCGG
CTCCACGTCGAGCCAGGGATAGGACAAAGGGTCCACTCACCCCGGATTTAAAACGCGTCTTTCCAACGCGCCGTCAAT
TGTAATTTCTCTCTTCTCTTCAATTTAGAATTTTTGGTCCATAGCGCCCAATATACTGGGGTTCTACAATGTAGGCCTG
GCCGTAACATCAGCCAATTTTGCTGTTTAACGATTATTGGAAAAAAGAATTTTCTTTTTTATAAAAACGCCAAATCGA
AAATATTTACAACAAAAATTATTTAATTTGGGTGGCGTATAACGGGGTTATTGTCACGGCCAGGCATACATTGGAGAA
CCTCAGTGTATTAGGCGCTATTAACGTTGCGTACCCCCTGTTCGGCACCCCCCCTGTTCGGCACCCTGAAAATCTA

Mai2 (*AvrPii*-C_{pro}/$J_{CDS+3'}$)

TCAGCGGATATTCACCAAAATAAGTAAATATTTAATTAAAAAAGCAAAGCGCAAATTTATAAAAAAAACATTAACATT
TCAAGTGAAGATAATTAAAGAAAAGGTAAATTAAAAGGCATAATAATTTCGTAAAAAGCGGTCTAATAAAATCTTTT
TTTACAAGATTGCGTCTTTATATAAATAGTAAATTATGCATTTGAGTTTTTTTGTTTCTATTAACAGTGGTACGGAGT
GGCGTGGCTAGGCGACTGCCAACCGTGACTCCACCCCACGTGGTAAGGTGCTAGGGTTCGTTGTTGTGTATTCGTTTA
GGCGCGTGCACGCGATTGGCATTGCGTGCCTTTCTTCTTTTTTTAGACTTAGCTTTAGATCTTCGAATAGAATCGCCC
CTATATTGCAACCTTGTCTCTTTGGTATCGCTTTCGTGACAAACAGAACTGTTTTTAAACAAGGTAATTAATTTGAAT

TTTGCACCTTTACGCGGACTTATGCAAACCGAAATCCCTAGGAAATGCTATTAATCATATAATATAAAAACCTCAACTT
CTTGTACACTACTTCGTTATATTTCCATTGCTATTCATCCTTTTTATTATAAAGCCAACGAATTGAATTCAGTTTTAGT
ATACTAAGATCCCCTTGTATTTTTTCCAACTTACCAAAATGCAACTTTCCAAAATTACTTTCGCTATTGCATTATATGC
AATCGGAATCGCAGCACTTCCCACTCCGGCCAGCCTGAATGGCAACACTGAGGTCGCAACCATCTCCGACGTTAAACT
TGAGGCCCGCAGCGACACCACTTATCATAAATGCTCCAAATGCGGTTATGGCAGCGATGATTCCGACGCGTATTTTAA
TCATAAATGCAACTAATGTAAGTTAAAATCTGAAAGCGCACGATTTAAATTAGTTTCAAATATAAATTATCTTAACGT
TTAATTATAGCGCATACAAAGTAAGCTGGATTTTTCCAAACGAACGGCGGACAGTTATGGACTATTGCGGGAATTTCA
AGGATAGGAAAAGCCAATTTGAAATATCCGGAATACTTTTTCCAAGCTTAACCATTAAATAACATGTCTTAAATCAAT
CTACCTTTCGCGTTTAAATTTTTTCAAACATAATGAACTGATATCAAAACCGAGGATGCTGCGAGCTGCGTTATGTTA
ATTTGTCGTTTTCTACACGATAGCGACTGATAATAGCAACAACACACATGTACATACACACATATACACATATATACCA
TTATTCCAAACACGTTAAGAACTGTGAAAAAATAGGTGGTTTTTATTTTGGCATGTTGGTAAAGCGTGAGAGCAGAG
TGGAGCAACCCTTGGATAATGGCTACTAAGGATATCCATCTGTGCATTATATAATAACGTGGATGTACACCGAGTCGCG
CAGGACACCGAGCGCGCACCCTTTATACATAGTAAATCCGTTCGTGTGCTTCGCCCAATTCAAGTCTAGTTTTCACCAA
AGTTCCAAATCTG

Mai3 (*AvrPii*-J/C-1)

TGGTAGATATCCGCTGACTGGGGGCAACATTGGTTGGATTTTAAAGTACATATATAGAAACGAAAATAAGTGGAAAAA
GAAGAACAAGAGTAAGGGTTATTAATTACACGACTAAATGTGGTAATTTAGCTATGTTATAACCAGGCAAGCACTACA
AATAGCCCATGGCAATCAGGAAGAGGCCGATATGTTACGATTGAATAATAAAATATTTTATAAACTTGCATTTGGATG
TAAATTGTAAATTATGCATTTGAGCTCCTCTGTTTCTATTAACAGAAATGTTTTTAAACAAGGTACCTAAATGCAATT
CTATATTTTTTATGCGGATTTATGCAGGCCCAAATCCGTAGGAAATACTATTAGTTATAATAATATAAAAACCTCGGCT
TCTTGTATATTACTGTATAATTCCTTTTCTTCCCTCCTTATATTTTTCATCTTAAAACTAAACATTTAAATTCAGCTTC
AAATATACCAAAATCCCCTTTTATTCCTTCCAATTTACCAAAATGCAACTCTCCAAAATTACTTTCGCTATTGCATTAT
ATGCAGTCGGAATCGCAGCACTTCCCACTCCGGCCAGCCTGAATGGCAACACTGAGGTCGCAACCATCTCCGACGTTA
AACTTGAGGCCCGCAGCGACACCACTTATCATAAATGCTCCAAATGCGGTTATGGCAGCGATGATTCCGACGCGTATT
TTAATCATAAATGCAACTAATGTAAGTTAAAATCTGAAAGCGCACGATTTAAATTAGTTTCAAATATAAATTATCTTA
ACGTTTAATTATAGCGCATACAAAGTAAGCTGGATTTTTCCAAACGAACGGCGGACAGTTATGGACTATTGCGGGAAT
TTCAAGGATAGGAAAAGCCAATTTGAAATATCCGGAATACTTTTTCCAAGCTTAACCATTAAATAACATGTCTTAAAT
CAATCTACCTTTCGCGTTTAAATTTTTTCAAACATAATGAACTGATATCAAAACCGAGGATGCTGCGAGCTGCGTTAT
GTTAATTTGTCGTTTTCTACACGATAGCGACTGATAATAGCAACAACACACATGTACATACACACATATACACATATAT

ACCATTATTCCAAACACGTTAAGAACTGTGAAAAAATAGGTGGTTTTTATTTTGGCATGTTGGTAAAGCGTGAGAGC
AGAGTGGAGCAACCCTTGGATAATGGCTACTAAGGATATCCATCTGTGCATTATATAATAACGTGGATGTACACCGAGT
CGCGCAGGACACCGAGCGCGCACCCTTTATACATAGTAAATCCGTTCGTGTGCTTCGCCCAATTCAAGTCTAGTTTTCA
CCAAAGTTCCAAATCTG

Mai4 (*AvrPii*-J/C-2)

TGGTAGATATCCGCTGACTGGGGGCAACATTGGTTGGATTTTAAAGTACATATATAGAAACGAAAATAAGTGGAAAAA
GAAGAACAAGAGTAAGGGTTATTAATTACACGACTAAATGTGGTAATTTAGCTATGTTATAACCAGGCAAGCACTACA
AATAGCCCATGGCAATCAGGAAGAGGCCGATATGTTACGATTGAATAATAAAATATTTTATAAACTTGCATTTGGATG
TAAATTGTAAATTATGCATTTGAGCTCCTCTGTTTCTATTAACAGAAATGTTTTTAAACAAGGTACCTAAATGCAATT
CTATATTTTTTATGCGGATTTATGCAGGCCCAAATCCGTAGGAAATACTATTAGTTATAATAATATAAAAACCTCGGCT
TCTTGTATATTACTGTATAATTCCTTTTCTTCCCTCCTTATATTTTTCATCTTAAAACTAAACATTTAAATTCAGCTTC
AAATATACCAAAATCCCCTTTTATTCCTTCCAATTTACCAAAATGCAACTCTCCAAAATTACTTTCGCTATTGCATTAT
ATGCAGTCGGAATCGCAGCACTTCCCACTCCGGCCTGCCTAAATGGCAACACTGAGGTCGCAACCATCTCCGACGTTA
AACTTGGACCCCGCAGCGACACCACTTATCATAAATGCTCCAAATGCGGTTATGGCAGCGATGATTCCGACGCGTATT
TTAATCATAAATGCAACTAATGTAAGTTAAAATCTGAAAGCGCACGATTTAAATTAGTTTCAAATATAAATTATCTTA
ACGTTTAATTATAGCGCATACAAAGTAAGCTGGATTTTTCCAAACGAACGGCGGACAGTTATGGACTATTGCGGGAAT
TTCAAGGATAGGAAAAGCCAATTTGAAATATCCGGAATACTTTTTCCAAGCTTAACCATTAAATAACATGTCTTAAAT
CAATCTACCTTTCGCGTTTAAATTTTTTCAAACATAATGAACTGATATCAAAACCGAGGATGCTGCGAGCTGCGTTAT
GTTAATTTGTCGTTTTCTACACGATAGCGACTGATAATAGCAACAACACACATGTACATACACACATATACACATATAT
ACCATTATTCCAAACACGTTAAGAACTGTGAAAAAATAGGTGGTTTTTATTTTGGCATGTTGGTAAAGCGTGAGAGC
AGAGTGGAGCAACCCTTGGATAATGGCTACTAAGGATATCCATCTGTGCATTATATAATAACGTGGATGTACACCGAGT
CGCGCAGGACACCGAGCGCGCACCCTTTATACATAGTAAATCCGTTCGTGTGCTTCGCCCAATTCAAGTCTAGTTTTCA
CCAAAGTTCCAAATCTG

Mai5 (*AvrPii*-J/C-3)

TGGTAGATATCCGCTGACTGGGGGCAACATTGGTTGGATTTTAAAGTACATATATAGAAACGAAAATAAGTGGAAAAA
GAAGAACAAGAGTAAGGGTTATTAATTACACGACTAAATGTGGTAATTTAGCTATGTTATAACCAGGCAAGCACTACA
AATAGCCCATGGCAATCAGGAAGAGGCCGATATGTTACGATTGAATAATAAAATATTTTATAAACTTGCATTTGGATG
TAAATTGTAAATTATGCATTTGAGCTCCTCTGTTTCTATTAACAGAAATGTTTTTAAACAAGGTACCTAAATGCAATT
CTATATTTTTTATGCGGATTTATGCAGGCCCAAATCCGTAGGAAATACTATTAGTTATAATAATATAAAAACCTCGGCT

TCTTGTATATTACTGTATAATTCCTTTTCTTCCCTCCTTATATTTTTCATCTTAAAACTAAACATTTAAATTCAGCTTC

AAATATACCAAAATCCCCTTTTATTCCTTCCAATTTACCAAAATGCAACTCTCCAAAATTACTTTCGCTATTGCATTAT

ATGCAGTCGGAATCGCAGCACTTCCCACTCCGGCCTGCCTAAATGGCAACACTGAGGTCGCAACCATCTCCGACGTTA

AACTTGGACCCCGCAGCGACAAGACTTATTATTATTGCTCCAAATGCGGCTTCGGCAGCGATGATTCCGACGCGTATT

TTAATCATAAATGCAACTAATGTAAGTTAAAATCTGAAAGCGCACGATTTAAATTAGTTTCAAATATAAATTATCTTA

ACGTTTAATTATAGCGCATACAAAGTAAGCTGGATTTTTCCAAACGAACGGCGGACAGTTATGGACTATTGCGGGAAT

TTCAAGGATAGGAAAAGCCAATTTGAAATATCCGGAATACTTTTTCCAAGCTTAACCATTAAATAACATGTCTTAAAT

CAATCTACCTTTCGCGTTTAAATTTTTTCAAACATAATGAACTGATATCAAAACCGAGGATGCTGCGAGCTGCGTTAT

GTTAATTTGTCGTTTTCTACACGATAGCGACTGATAATAGCAACAACACACATGTACATACACACATATACACATATAT

ACCATTATTCCAAACACGTTAAGAACTGTGAAAAAATAGGTGGTTTTTATTTTGGCATGTTGGTAAAGCGTGAGAGC

AGAGTGGAGCAACCCTTGGATAATGGCTACTAAGGATATCCATCTGTGCATTATATAATAACGTGGATGTACACCGAGT

CGCGCAGGACACCGAGCGCGCACCCTTTATACATAGTAAATCCGTTCGTGTGCTTCGCCCAATTCAAGTCTAGTTTTCA

CCAAAGTTCCAAATCTG

Mai6 (*AvrPii*-$J_{pro+CDS}$/$C_{3'}$)

TGGTAGATATCCGCTGACTGGGGGCAACATTGGTTGGATTTTAAAGTACATATATAGAAACGAAAATAAGTGGAAAAA

GAAGAACAAGAGTAAGGGTTATTAATTACACGACTAAATGTGGTAATTTAGCTATGTTATAACCAGGCAAGCACTACA

AATAGCCCATGGCAATCAGGAAGAGGCCGATATGTTACGATTGAATAATAAAATATTTTATAAACTTGCATTTGGATG

TAAATTGTAAATTATGCATTTGAGCTCCTCTGTTTCTATTAACAGAAATGTTTTTAAACAAGGTACCTAAATGCAATT

CTATATTTTTTATGCGGATTTATGCAGGCCCAAATCCGTAGGAAATACTATTAGTTATAATAATATAAAAACCTCGGCT

TCTTGTATATTACTGTATAATTCCTTTTCTTCCCTCCTTATATTTTTCATCTTAAAACTAAACATTTAAATTCAGCTTC

AAATATACCAAAATCCCCTTTTATTCCTTCCAATTTACCAAAATGCAACTTTCCAAAATTACTTTCGCTATTGCATTAT

ATGCAATCGGAATCGCAGCACTTCCCACTCCGGCCAGCCTGAATGGCAACACTGAGGTCGCAACCATCTCCGACGTTA

AACTTGAGGCCCGCAGCGACACCACTTATCATAAATGCTCCAAATGCGGTTATGGCAGCGATGATTCCGACGCGTATT

TTAATCATAAATGCAACTAATGTAAGTTGATATCTAAACGCATAATTTAACTCATTTTTAAAAAAATAAATTATTCTAA

CGTTTAATTATAATGCATACGAATTAAACAGCATTTTTCAAGCGCGTGACGGACAGTTATGGATTCTGCAAATTTTAA

ATACCCGGTGTCACGGCCAGGCATATATCGGAATGTGAGGATCAGGTCCCCACACCTACCCTCAGAATTACGACTCGGC

TCCACGTCGAGCCAGGGATAGGACAAAGGGTCCACTCACCCCGGATTTAAAACGCGTCTTTCCAACGCGCCGTCAATT

GTAATTTCTCTCTTCTCTTCAATTTAGAATTTTTGGTCCATAGCGCCCAATATACTGGGGTTCTACAATGTAGGCCTGG

CCGTAACATCAGCCAATTTTGCTGTTTAACGATTATTGGAAAAAAGAATTTTCTTTTTTATAAAAACGCCAAATCGAA

AATATTTACAACAAAAATTATTTAATTTGGGTGGCGTATAACGGGGTTATTGTCACGGCCAGGCATACATTGGAGAAC

CTCAGTGTATTAGGCGCTATTAACGTTGCGTACCCCCTGTTCGGCACCCCCCCTGTTCGGCACCCTGAAAATCTA

Mai7 (*AvrPii*-C$_{pro+CDS}$/J$_{3'}$)

TCAGCGGATATTCACCAAAATAAGTAAATATTTAATTAAAAAAGCAAAGCGCAAATTTATAAAAAAAACATTAACATT

TCAAGTGAAGATAATTAAAGAAAAGGTAAATTAAAAGGCATAATAATTTCGTAAAAAGCGGTCTAATAAAATCTTTT

TTTACAAGATTGCGTCTTTATATAAATAGTAAATTATGCATTTGAGTTTTTTTGTTTCTATTAACAGTGGTACGGAGT

GGCGTGGCTAGGCGACTGCCAACCGTGACTCCACCCCACGTGGTAAGGTGCTAGGGTTCGTTGTTGTGTATTCGTTTA

GGCGCGTGCACGCGATTGGCATTGCGTGCCTTTCTTCTTTTTTTAGACTTAGCTTTAGATCTTCGAATAGAATCGCCC

CTATATTGCAACCTTGTCTCTTTGGTATCGCTTTCGTGACAAACAGAACTGTTTTTAAACAAGGTAATTAATTTGAAT

TTTGCACCTTTACGCGGACTTATGCAAACCGAAATCCCTAGGAAATGCTATTAATCATATAATATAAAAACCTCAACTT

CTTGTACACTACTTCGTTATATTTCCATTGCTATTCATCCTTTTTATTATAAAGCCAACGAATTGAATTCAGTTTTAGT

ATACTAAGATCCCCTTGTATTTTTTCCAACTTACCAAAATGCAACTCTCCAAAATTACTTTCGCTATTGCATTATATGC

AGTCGGAATCGCAGCACTTCCCACTCCGGCCTGCCTAAATGGCAACACTGAGGTCGCAACCATCTCCGACGTTAAACT

TGGACCCCGCAGCGACAAGACTTATTATTATTGCTCCAAATGCGGCTTCGGCAGCAATGATTCCGACACGTATTTTAA

TCACAAGTGCAAGTAATGTAAGTTAAAATCTGAAAGCGCACGATTTAAATTAGTTTCAAATATAAATTATCTTAACGT

TTAATTATAGCGCATACAAAGTAAGCTGGATTTTTCCAAACGAACGGCGGACAGTTATGGACTATTGCGGGAATTTCA

AGGATAGGAAAAGCCAATTTGAAATATCCGGAATACTTTTTCCAAGCTTAACCATTAAATAACATGTCTTAAATCAAT

CTACCTTTCGCGTTTAAATTTTTTCAAACATAATGAACTGATATCAAAACCGAGGATGCTGCGAGCTGCGTTATGTTA

ATTTGTCGTTTTCTACACGATAGCGACTGATAATAGCAACAACACACATGTACATACACACATATACACATATATACCA

TTATTCCAAACACGTTAAGAACTGTGAAAAAATAGGTGGTTTTTATTTTGGCATGTTGGTAAAGCGTGAGAGCAGAG

TGGAGCAACCCTTGGATAATGGCTACTAAGGATATCCATCTGTGCATTATATAATAACGTGGATGTACACCGAGTCGCG

CAGGACACCGAGCGCGCACCCTTTATACATAGTAAATCCGTTCGTGTGCTTCGCCCAATTCAAGTCTAGTTTTCACCAA

AGTTCCAAATCTG
