# Supplementary material for: The Lesson Learned from the Unique Evolutionary Story of Avirulence Gene AvrPii of Magnaporthe oryzae
Source: Genes (Basel). 2023 May 11;14(5):1065. doi: 10.3390/genes14051065 (PMC10218241; doi:10.3390/genes14051065)
Supplement: Supplementary file 1 [file genes-14-01065-s001.zip › genes-2373462-supplementary/23-5-7 Supplementary Materials for AvrPii/Table S4. Comparison of evolutionary parameters.pdf]

Table S4. Comparison of evolutionary parameters of the *AvrPii* family among three *Mo* populations, of which parameters of the two populations previously reported were re-computed by the same approaches used in the current study

**Population**	S [a]	π [b]	D^* [c]	F^*	Ka [d]	Ks [e]	Ka/Ks
**All *AvrPii* (the current study)**							
Entire coding region	20	0.037	1.74 $^{\beta}$	2.65 $^{\beta}$	193.8	357.6	0.54
Non-signal peptide region	18	0.045	1.68 $^{\alpha}$	2.59 $^{\beta}$	246.8	434.8	0.57
Signal peptide region	2	0.013	0.68	1.11	54.9	166.0	0.33
**All *AvrPii* (Lu et al. 2019)**							
Entire coding region	15	0.013	1.58 $^{\alpha}$	1.05	19.80	22.39	0.88
Non-signal peptide region	14	0.017	1.55 $^{\alpha}$	1.04	24.97	31.48	0.79
Signal peptide region	1	0.003	0.54	0.34	5.98	0	Ka>Ks
**All *AvrPii* (Sirisathaworn et al. 2017)**							
Entire coding region	11	0.003	-3.81 $^{\beta}$	-3.92 $^{\beta}$	3.71	0.88	4.23
Non-signal peptide region	5	0.002	-2.20	-2.48	3.07	0	Ka>Ks
Signal peptide region	6	0.004	-3.90 $^{\beta}$	-3.90 $^{\beta}$	5.49	3.33	1.65

[a] Number of segregating sites.

[b] Nei's nucleotide diversity based on silent site.

[c] Fu and Li's D^* and F^*, and $^{\alpha}$, $^{\beta}$ represents statistical significance at $P < 0.05$, 0.02 level, respectively.

[d] Nonsynonymous.

[e] Synonymous.
